# A Pesticide Decision Support Tool to guide the selection of less environmentally harmful pesticides for the sugar cane industry

**DOI:** 10.1007/s11356-023-29814-w

**Published:** 2023-09-25

**Authors:** Michael St. John Warne, Peta A. Neale, Michael J. Macpherson

**Affiliations:** 1 Reef Catchments Science Partnership, School of the Environment, University of Queensland, Brisbane, QLD 4108 Australia; 2 Water Quality and Investigations, Environmental Monitoring and Assessment Science, Science Delivery, Department of Environment and Science, Brisbane, Queensland 4102 Australia; 3 Centre for Agroecology, Water and Resilience, Coventry University, Coventry, UK; 4 Farmacist Pty Ltd, Mackay, Queensland Australia

**Keywords:** Pesticide active ingredients, Aquatic risk, Mobility, Persistence, Toxicity, Sugar cane

## Abstract

Pesticides applied to agricultural land have been shown to decrease the quality of water entering the Great Barrier Reef lagoon. This issue is addressed by the Reef 2050 Water Quality Improvement Plan which includes a pesticide reduction target. As part of a wider educational strategy, one method that could help meet the target is to provide stakeholders with information that assists in the selection and use of pesticide active ingredients (PAIs) that pose a lower risk to aquatic environments compared to those currently used. This study developed a Pesticide Decision Support Tool (PDST) in collaboration with stakeholders for the sugar cane industry. The PDST covers all PAIs registered and applied to sugar cane in Australia and four additional PAIs registered for use on crops grown in rotation with sugar cane. The PDST incorporates both the measure of mobility and persistence of a PAI and the measure of effect, which is based on the PAI application rate and ecotoxicity threshold value. The aquatic risk, which is the product of the measure of effect and the measure of mobility and persistence, is a measure of the likelihood that a PAI will reach the aquatic environment and cause harmful effects. Insecticide active ingredients (e.g., cadusafos, chlorpyrifos) posed the greatest aquatic risk, followed by herbicide active ingredients (e.g., MSMA, metolachlor), while fungicide AIs typically had a lower aquatic risk. An interactive spreadsheet allows characteristics, including application rate and tank mixes, to be considered when assessing the potential risk. While focusing on sugar cane, the results are equally appropriate to other crops that use the same PAIs provided the application rates are corrected to the new crop. In addition, the approach used in the PDST can be applied internationally and to any PAIs with sufficient toxicity, mobility, and persistence data.

**Supplementary Information:**

The online version contains supplementary material available at 10.1007/s11356-023-29814-w.

## Introduction

Great Barrier Reef (GBR) ecosystems are in decline, with suspended sediment, nutrients, and pesticides identified as the main factors reducing water quality (Brodie et al. 2019). Despite comprising only around 1% of the total land in the GBR catchments, it has been demonstrated that sugar cane cultivation is one of the main contributors to the pesticide load. This is particularly the case in the Wet Tropics, Burdekin, Mackay Whitsunday, and Burnett Mary natural resource management (NRM) regions (Bartley et al. 2017), where the area devoted to sugar cane production comprises a larger percentage of the catchment land use (Warne et al. 2020). A recent study on the pesticide toxicity hazard in Australia identified the sugar cane-growing areas of Far North Queensland as aquatic ecotoxicity hazard hotspots (Navarro et al. 2021).

Being much closer to the point of pesticide application (i.e., agriculture), freshwater environments experience a greater risk from pesticide active ingredients (PAIs) than ecosystems further away such as nearshore or offshore marine environments (Brodie and Landos 2019). For example, O'Brien et al. (2016) found that concentrations of photosystem II (PSII) herbicide AIs ametryn, atrazine, and diuron frequently exceeded the proposed guideline values in Barratta Creek in the Burdekin region. Further, diuron and metolachlor concentrations exceeded guideline values in 84 and 53% of water samples collected from Sandy Creek in the Mackay Whitsunday region, respectively (Wallace et al. 2017). Lower PAI concentrations were detected in nearshore environments in the Wet Tropics compared to the river mouths, with no PAIs detected in mid- and outer-shelf reefs (Shaw et al. 2010).

Efforts to reduce pesticide inputs into the aquatic environment have primarily focused on the regulation of identified problem PAIs, improved farm management practices, and new application techniques. An additional complementary approach is to substitute problematic PAIs with alternative PAIs that pose a lower risk to aquatic environments. Factors involved in selecting lower risk PAIs include toxicity, sorption to soil, loss to runoff, application rate, and environmental half-life (Silburn et al. 2023). While there is a shift away from the use of traditional PSII herbicides such as diuron (driven by regulation) in the GBR catchments, many of the alternative herbicides being used as substitutes may pose a similar environmental risk (Davis et al. 2014). Consequently, there is a need for a simple tool to assess and compare the aquatic risk of PAIs registered for use on sugar cane.

Pesticide risk indicators are tools that can help support decision-making by assessing the risks associated with pesticide use (Kookana and Oliver 2018). There are a number of pesticide risk indicators currently available, and these range from very simple tools with few input parameters to complex models (e.g., Padovani et al. 2004; Kookana et al. 2005; Juraske et al. 2007; Le Bellec et al. 2015; Strassemeyer et al. 2017; Astaykina et al. 2020). Data availability is one limitation of the more complex pesticide risk indicators. Consequently, some indicators assess risk based on readily available physicochemical properties and the toxicity of the PAIs alone (e.g., Kudsk et al. 2018), while others also incorporate site-specific characteristics, such as rainfall, slope, and temperature (e.g., Kookana et al. 2005; Le Bellec et al. 2015). Pesticide risk indicators that include exposure-toxicity ratios (i.e., the ratio between the PAI concentration in the environment and the toxicity of the PAI to relevant organisms) are considered more suitable for estimating the environmental risk than those that do not (Feola et al. 2011). Several pesticide risk indicators use the organic carbon-water partition coefficient (*K*_OC_) as an indicator of pesticide transport in runoff (Kookana et al. 2005; Dabrowski and Balderacchi 2013). Further, many indicators apply acute toxicity data to evaluate the toxicity to aquatic organisms (e.g., Kookana et al. 2005; Juraske et al. 2007; Le Bellec et al. 2015), though chronic toxicity data are more suitable for estimating the longer-term risk from persistent ongoing exposure to levels of PAIs below acute toxicity thresholds. In order for pesticide risk indicators to be useful in a policy development and assessment context, Maud et al. (2001) developed a list of desirable characteristics, which included using readily available data, being simple and transparent, avoiding controversial weighting schemes, and being able to clearly differentiate between different products.

In the current study, we developed a simple Pesticide Decision Support Tool (PDST) to assist famers, agronomists, and resellers select PAIs that pose a lower ecological risk in receiving waters. The PDST included both the measure of mobility and persistence of a PAI and the measure of effect, which was based on the PAI application rate and inherent toxicity using ecotoxicity thresholds. The ecotoxicity thresholds were derived using chronic toxicity data and either species sensitivity distributions or the assessment factor method. Data from freshwater species were preferentially used to derive the ecotoxicity thresholds given that freshwater species are more likely to be exposed to higher pesticide concentrations than marine species, as freshwater environments are the primary receptor for agricultural runoff in GBR catchments. The PDST does not attempt to predict the concentration of a PAI in the aquatic environment at any individual point, but rather estimates the likelihood that a PAI will reach a waterway and cause harmful effects. The current study included 47 PAIs registered for use on sugar cane or crops grown in rotation with sugar cane or that are widely used in sugar cane-growing regions in Queensland, Australia.

## Methods

### Stakeholder engagement

The PDST was developed in consultation with stakeholders from government and key pesticide users including farmers, resellers, extension officers, and agronomists. Twenty-three stakeholder meetings were held during 2019 and early 2020 to obtain information about relevant pesticide usage, key factors to consider, how simple or complex the PDST should be, the best format for presenting the information, and whether draft versions of the PDST were understandable, simple to use, and fit for purpose.

### Active ingredient selection

A total of 47 PAIs were included in the PDST (Table S1 of the Electronic Supplementary Material (ESM)). The majority are registered for use in sugar cane and were selected by searching the Public Chemical Registration Information System (PubCRIS) (APVMA 2019), which is maintained by the Australian Pesticides and Veterinary Medicines Authority (APVMA). The list was screened by industry experts to identify PAIs currently applied to sugar cane, resulting in 42 PAIs. While wetting agents, adjuvants, and metallic PAIs are also registered for use in sugar cane, they were not included in the current study due to the general lack of appropriate fate and effect data. In Queensland, Australia, sugar cane is often grown in rotation with crops such as mung bean, soybean, corn (maize), and rice. Therefore, four PAIs used on these rotation crops, chlorothalonil, fluazifop-P-butyl, haloxyfop, and terbuthylazine, were also included. Finally, metsulfuron-methyl was included as it is used by sugar mills for weed control of cane train corridors (Rob Sluggett, *pers. comm*). Of the 47 PAIs in the PDST, 32 are herbicides, 9 are insecticides, 5 are fungicides, and 1 is a nematicide (Table 1). All of the 47 PAI included in the PDST are currently registered for use in Australia.

**Table 1:** The proposed freshwater default guideline values (DGVs) and ecotoxicity threshold values (ETVs) for the studied pesticide active ingredients and the method used to derive them and their reliability

Pesticide active ingredient	Chemical class	Limit used (μmol/L)	Type of limit	Derivation method (reliability)
2,4-D	Herbicide	0.19	ETV (current study)	SSD (high)
Ametryn	Herbicide	0.00044	Proposed DGV (King et al. 2017a)	SSD (high)
Amicarbazone	Herbicide	0.0083	ETV (current study)	SSD (low)
Asulam	Herbicide	0.94	ETV (current study)	SSD (moderate)
Atrazine	Herbicide	0.018	Proposed DGV (DES 2019)	SSD (very high)
Bifenthrin	Insecticide	0.00000043	ETV (current study)	SSD (low)
Cadusafos	Insecticide	0.0000067	ETV (current study)	AF (unknown)
Carbofuran	Insecticide	0.0090	ETV (current study)	SSD (very high)
Chlorothalonil	Fungicide	0.0018	Proposed DGV (King et al. 2017b)	SSD (high)
Chlorpyrifos	Insecticide	0.00000013^a^	ETV (current study)	SSD (very high)
Clothianidin	Insecticide	0.0012	ETV (Spilsbury 2018)	SSD (low)
Dicamba	Herbicide	0.28	ETV (Oekotoxzentrum 2020)	SSD (high)
Diquat dibromide	Herbicide	0.0032	ETV (current study)	SSD (low)
Diuron	Herbicide	0.0030	Proposed DGV (King et al. 2017a)	SSD (high)
Fipronil	Insecticide	0.000020^a^	ETV (current study)	SSD (moderate)
Fluazifop-P-butyl	Herbicide	0.012^a,b^	ETV (current study)	AF (unknown)
Fluensulfone	Nematicide	0.041	ETV (current study)	SSD (moderate)
Flumioxazin	Herbicide	0.000012	ETV (current study)	SSD (moderate)
Fluroxypyr	Herbicide	1.2	Proposed DGV (King et al. 2017b)	SSD (moderate)
Flutriafol	Fungicide	0.79	ETV (current study)	SSD (low)
Glufosinate ammonium	Herbicide	0.48	ETV (current study)	SSD (low)
Glyphosate	Herbicide	1.5	Proposed DGV (King et al. 2017a)	SSD (moderate)
Halosulfuron-methyl	Herbicide	0.00011	ETV (current study)	SSD (low)
Haloxyfop	Herbicide	5.5	Proposed DGV (King et al. 2017b)	SSD (low)
Hexazinone	Herbicide	0.0044	Proposed DGV (King et al. 2017a)	SSD (low)
Imazapic	Herbicide	0.0015	Proposed DGV (King et al. 2017a)	SSD (very low)
Imidacloprid	Insecticide	0.00047	Proposed DGV (King et al. 2017a)	SSD (moderate)
Isoxaflutole	Herbicide	0.0013	Proposed DGV (King et al. 2017a)	SSD (low)
MCPA	Herbicide	0.011	ETV (Spilsbury 2018)	SSD (very high)
Metolachlor	Herbicide	0.0016	Proposed DGV (King et al. 2017a)	SSD (very high)
Metribuzin	Herbicide	0.012	Proposed DGV (King et al. 2017a)	SSD (high)
Metsulfuron-methyl	Herbicide	0.000063	Proposed DGV (King et al. 2017a)	SSD (moderate)
MSMA	Herbicide	0.0050	ETV (current study)	AF (unknown)
Paraquat dichloride	Herbicide	0.011	Proposed DGV (DES 2019)	SSD (moderate)
Pendimethalin	Herbicide	0.00017^a^	Proposed DGV (King et al. 2017b)	SSD (moderate)
Permethrin	Insecticide	0.000026^a^	Proposed DGV (DES 2019)	SSD (moderate)
Picloram	Herbicide	2.3	Proposed DGV (DES 2019)	SSD (low)
Propiconazole	Fungicide	0.029	Proposed DGV (King et al. 2017b)	SSD (moderate)
S-Metolachlor	Herbicide	0.00081	ETV (current study)	SSD (low)
Tebuconazole	Fungicide	0.017	ETV (Oekotoxzentrum 2020)	SSD (moderate)
Terbuthylazine	Herbicide	0.0052	Proposed DGV (King et al. 2017b)	SSD (very high)
Terbutryn	Herbicide	0.0015	Proposed DGV (King et al. 2017b)	SSD (moderate)
Triadimenol	Fungicide	0.41	ETV (current study)	SSD (moderate)
Trichlorfon	Insecticide	0.00013	ETV (current study)	SSD (very high)
Trifloxysulfuron sodium	Herbicide	0.00081	ETV (current study)	SSD (low)
Trifluralin	Herbicide	0.0000098^a^	ETV (current study)	SSD (moderate)
Trinexapac-ethyl	Herbicide	0.37	ETV (current study)	SSD (low)

*AF* assessment factor, *SSD* species sensitivity distribution

^a^These chemicals have log *K*_OW_ values greater than four and are classed as potential biomagnifiers. Therefore, we have used PC99 for these chemicals, rather than PC95 as recommended in Warne et al. (2018)

^b^These ETVs could not be adjusted for biomagnification as they were not derived using the species sensitivity distribution method

### Active ingredient application rates

Pesticide labels for each PAI were collected from PubCRIS (APVMA 2019). The concentration of PAI in the product, usually in units of g/kg or g/L, was expressed as a percentage. The minimum and maximum product application rates to sugar cane in units of kg/ha or L/ha were obtained from the label, with the PAI application rate determined by multiplying the product application rate and PAI concentration percentage. This data determines the concentration of each PAI applied to a unit of land. The insecticide imidacloprid is used in both liquid and slow release pellet forms, with the latter being applied once every 4 years (although until recently its application rate was once every 3 years). Both forms of imidacloprid were considered in the PDST, with the slow-release application rate divided by four to obtain an estimated annual application rate to facilitate comparison with the liquid form as the formulation is specifically designed (and tested) to release an equal dosage of imidacloprid each year for a 4-year period. In the case of the PAIs used for rotation crops, the application rates to sweet corn were used for chlorothalonil, while the application rates to soybeans were used for fluazifop-P-butyl, haloxyfop, and terbuthylazine. Of the four rotation crops, chlorothalonil can only be applied to corn, fluazifop-P-butyl can only be applied to soybeans, and haloxyfop and terbuthylazine can both be applied to soybean and mung bean at similar application rates. A representative application rate of 0.005 to 0.007 kg of PAI per hectare (kg PAI/ha) was used for metsulfuron-methyl (Allan Blair, *pers. comm.*). Product and PAI application rates and concentration percentage for each PAI are provided in Table S2, ESM.

### Physicochemical properties

Physicochemical properties of the selected PAIs including solubility, the organic carbon-water partition coefficient (*K*_OC_), acid dissociation constant (pK_a_), degradation half-life in soil and water, and octanol-water partition coefficient (*K*_OW_) were collected from the Pesticide Properties Database (PPDB) (University of Hertfordshire 2013) and US EPA EPI Suite (US EPA 2012) (Table S1). As experimental *K*_OC_ values were not available for all PAIs in the PPDB, *K*_OC_ values predicted using the OPEn structure–activity Relationship App (OPERA) model were collected from the US EPA CompTox Chemistry Dashboard (US EPA 2019c).

### Water quality guideline values and ecotoxicity threshold values

Proposed freshwater Australian and New Zealand default guideline values (DGVs) or ecotoxicity threshold values (ETVs) were used as the ecotoxicity thresholds for the PAIs. Proposed DGVs were obtained from the Queensland Department of Environment and Science (DES) (DES 2019), with earlier versions of the proposed DGVs (available in King et al. (2017a) and King et al. (2017b)). In cases where proposed DGVs were not available, ETVs were derived as part of this study using the same methodology proposed by Warne et al. (2018) for establishing DGVs. Briefly, this involved collating ecotoxicity data from the ECOTOX Knowledgebase (US EPA 2019a) and the OPP (Office of the Pesticide Program) Pesticide Ecotoxicity database (US EPA 2019b) and then screening and evaluating the quality of the data using the aforementioned methodology. Acceptable data were used to calculate a single toxicity value for each species, which were then used to generate a species sensitivity distribution using the Burrlioz V2 (CSIRO 2016) software package to determine the concentration that should protect 99%, 95%, 90%, and 80% of species (i.e., PC99, PC95, PC90, and PC80). PC95 values were used for the PDST, with the exception of PAIs with a log *K*_OW_ of 4 or greater, where the PC99 was used as recommended by Warne et al. (2018) due to the potential of these PAIs to bioaccumulate (ANZECC and ARMCANZ 2000). In cases where there were not sufficient acceptable toxicity data, ETVs were estimated using the less reliable Australian and New Zealand Assessment Factor method (Warne 2001). Briefly, this involved selecting the single lowest toxicity value and dividing it by an appropriate assessment factor. All ETVs were expressed as μmol/L, as this allows the toxicity of the PAIs to be compared and ranked.

### Measure of effect

The measure of effect indicates the potential of a PAI to exert harmful effects on aquatic organisms and incorporates the inherent toxicity of the PAI and the amount of that PAI that can be applied to an area of land for the purposes of pest management in the relevant cropping situation. The measure of effect for each PAI can be considered a surrogate for an exposure to toxicity ratio and was calculated using Eq. 1:

1 $$\text{Measure}\;\text{of}\;\text{effect}=\frac{\text{Maximum}\;\text{application}\;\text{rate}}{{\mathrm{DGV}}_{\mathrm{PAI}}\;\text{or}\;{\mathrm{ETV}}_{\mathrm{PAI}}}$$

where DGV_PAI_ is the default guideline value for the PAI and ETV_PAI_ is the ecotoxicity threshold value for the PAI in μmol/ML. The maximum application rate is in μmol PAI/ha. Further information about the PAI application rates and DGVs/ETVs can be found in the sections above.

### Measure of mobility and persistence

**Fig. 1:**
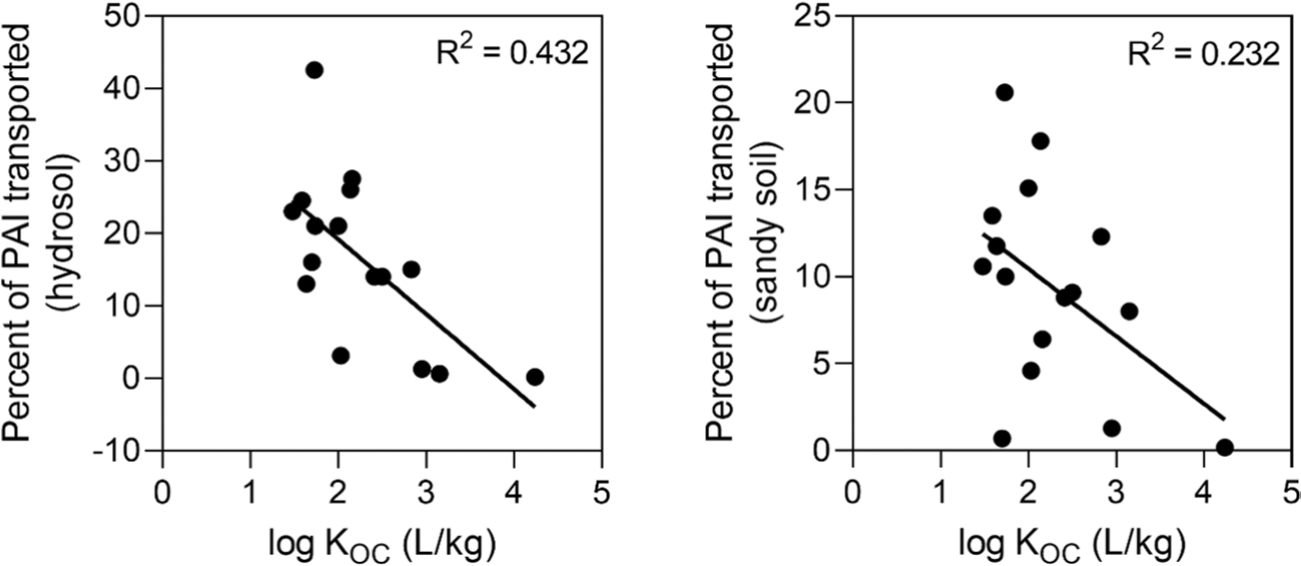
Linear regression of log *K*_OC_ and experimental percent of PAI transported for hydrosol and sandy soil

The measure of mobility and persistence indicates the potential of a PAI to move from farmland into the aquatic environment via runoff and to persist in that environment. *K*_OC_ was used as an indicator of mobility. To evaluate the suitability of *K*_OC_ for predicting mobility, experimental runoff loss data from field trials from the Wet Tropics region (Fillols et al. 2018) were compared with *K*_OC_. The experimental runoff data were for two different soil types, a well-drained deep sandy soil and a poorly drained hydrosol soil, both with a green cane trash blanket with simulated rainfall applied 2 days after pesticide application. The PAIs were ranked in terms of their mobility (e.g., proportion of PAI loss) in the sandy soil and hydrosol, and each was then compared with the rank of the corresponding *K*_OC_ values using a Spearman rank correlation coefficient test. For both soil types, there was a negative trend between ranked experimental PAI loss and ranked *K*_OC_ (Figure S1), but the correlation was only statistically significant for the hydrosol (*r* = − 0.592, *P* = 0.017). However, when negatively charged MCPA was removed from the sandy soil comparison, the correlation coefficient between runoff loss and *K*_OC_ became significant (*r* = − 0.607, *P* = 0.019). Weak linear relationships, with *R*^2^ values of 0.432 and 0.232, were observed between log *K*_OC_ values and PAI losses in hydrosols and sandy soil, respectively (Fig. 1). The relationship between log *K*_OC_ and experimental runoff loss from the hydrosol soil was used to predict the percent of pesticide transported from soil for each PAI (Eq. 2):

2 $$\textrm{Percent}\ \textrm{of}\ \textrm{PAI}\ \textrm{transported}=-10.076\times \log\ {\textrm{K}}_{\textrm{OC}}+39.123$$

Percent of PAI transported was converted to proportion of PAI transported by dividing by 100. The relationship between *K*_OC_ and proportion of PAI transported is curvilinear and does not assume that the gradient equals 1.

Degradation half-life in soil and water was used as a surrogate for persistence. The measure of mobility and persistence was calculated as the product of the soil half-life (*t*_1/2 soil_), the aqueous phase half-life (*t*_1/2 water_), and the proportion of pesticide transported from soil (Eq. 3):

3 $$\textrm{Measure}\ \textrm{of}\ \textrm{mobility}\ \textrm{and}\ \textrm{persistence}={t}_{1/2\ \textrm{soil}}\times {t}_{1/2\ \textrm{water}}\times \textrm{proportion}\ \textrm{of}\ \textrm{PAI}\ \textrm{transported}$$

## Results and discussion

### Measure of effect

Of the 47 PAIs included in the PDST, proposed DGVs were available for 21. The Swiss have developed chronic water quality standards for dicamba and tebuconazole (Oekotoxzentrum 2020), and the data used to develop these standards were extracted to generate ETVs using the Australian and New Zealand derivation method (Warne et al. 2018). Further, Spilsbury (2018) derived ETVs for clothianidin and MCPA using the method of Warne et al. (2018). ETVs were derived for the other 22 PAIs as part of the current study (Section S1 of the ESM). For 19 of the 22 PAIs, there were sufficient toxicity data (i.e., for at least five species from four taxonomic groups) available to derive ETVs using the preferred species sensitivity distribution method (Warne et al. 2018). The remaining three PAIs, cadusafos, fluazifop-P-butyl, and MSMA, did not have sufficient ecotoxicity data to use the species sensitivity distribution approach, so the Australian and New Zealand assessment factor method (Warne 2001) was used to derive their ETVs.

All proposed DGVs or ETVs used in this study are listed in Table 1, along with their derivation method and reliability. Their reliability was determined using the method in Warne et al. (2018) which considers the type of toxicity data (chronic or acute), the number of phyla and species there are data available for, and the fit of the species sensitivity distribution to the toxicity data. The method classifies each DGV and ETV as having a very low, low, moderate, high, or very high reliability. Insecticide AIs bifenthrin, cadusafos, and chlorpyrifos were the most toxic, though it should be noted that chlorpyrifos and bifenthrin have a log *K*_OW_ greater than 4, so the PC99 was used for the ETV, rather than the PC95 for non-bioaccumulating PAIs. There was also very limited toxicity data available for cadusafos, so the ETV was derived using the assessment factor method, which is less scientifically rigorous than the species sensitivity distribution method, and ETVs derived using this method are often more conservative (lower) than values derived by the species sensitivity distribution method (Warne et al. 2018).

A set of DGVs or ETVs (PC99, PC95, PC90, and PC80) was derived for each PAI from chronic or converted acute toxicity data to protect against adverse effects from long-term exposure. In contrast, many other pesticide risk indicators use acute toxicity values to determine the risk to individual aquatic species, such as fish, daphnids, and algae (e.g., Juraske et al. 2007; Kookana and Oliver 2018). Dabrowski and Balderacchi (2013) used species sensitivity distributions to derive toxicity values for their predicted relative risk (PRRI) indicator, but this was also based on acute data. The toxicity data used in deriving the species sensitivity distribution and PC values for each PAI in this study were selected depending on whether the data were unimodal or bimodal. If the data were unimodal toxicity data for all species were used, whereas if the data were bimodal, then only the toxicity data for the most sensitive group of organisms were used (Warne et al. 2018). Which groups of organisms were compared depended on the mode of action of the PAI. For example, for an insecticide AI, the sensitivity of arthropods (including insects) would be compared to all other organisms, and for a herbicide AI, the sensitivity of phototrophs would be compared to all other organisms. With bimodal data, this approach ensures that the PC values provide the desired level of protection to the most sensitive group of organisms.

The potential of a PAI to induce an adverse effect will depend on both its inherent toxicity and the exposure concentration. The application rate of each PAI was used as a surrogate for exposure, with the maximum permitted application rate selected as the default value to permit a uniform comparison of the risk for all PAIs. A broadcast or blanket spray regime, where the PAI is applied to the whole hectare, was also assumed in the measure of effect calculations.

The measure of effect values ranged from 0.04 for the herbicide AI haloxyfop to 21,276,596 for the insecticide AI chlorpyrifos (Table 2). There was approximately a 2,000,000-fold variation in measure of effect values for herbicide AIs, a 14,000-fold variation in measure of effect values for insecticide AIs, and a 14,000-fold variation in measure of effect values for fungicide AIs. The application rates had a significant effect on the measure of effect values. This is illustrated by the herbicide AI metsulfuron-methyl, which was the seventh most toxic PAI based on its ETV but was ranked 30th based on measure of effect due its low maximum application rate (0.007 kg PAI/ha). In contrast, herbicide AI MSMA was the 24th most toxic PAI based on its ETV, but as it had the highest maximum application rate of any of the studied PAIs (4.75 kg PAI/ha), it was ranked 11th based on measure of effect.

**Table 2:** The measure of mobility and persistence, measure of effect (based on the maximum application rate), and aquatic risk for the sugar cane active ingredients. Pesticide active ingredients with a lower aquatic risk are less environmentally harmful, while pesticide active ingredients with a higher aquatic risk are more environmentally harmful. NB: values have been rounded off

Pesticide active ingredient	Measure of mobility and persistence	Measure of effect (maximum)	Aquatic risk (× 100)
2,4-D	18	52	9
Ametryn	155	20,000	31,000
Amicarbazone	305	350	1100
Asulam	48	16	8
Atrazine	1138	782	8900
Bifenthrin	0	208,333	21
Cadusafos	182	2,197,802	4,000,000
Carbofuran	35	1500	520
Chlorothalonil	0.13	3450	5
Chlorpyrifos	4	21,276,596	850,000
Clothianidin	3938	1724	68,000
Dicamba	87	5	4
Diquat dibromide	0	335	< 1
Diuron	136	2478	3400
Fipronil	777	5618	43,600
Fluazifop-P-butyl	40	48	20
Fluensulfone	26	160	40
Flumioxazin	4	85,366	3500
Fluroxypyr	27	2	1
Flutriafol	9977	1	100
Glufosinate ammonium	6	10	1
Glyphosate	12	13	2
Halosulfuron - methyl	40	2074	830
Haloxyfop	22	0.04	< 1
Hexazinone	1274	480	6100
Imazapic	6	234	15
Imidacloprid (L)	1046	2083	43,600
Imidacloprid (SR)	1046	4167	21,800
Isoxaflutole	0.06	326	< 1
MCPA	71	494	350
Metolachlor	1170	3757	44,000
Metribuzin	168	600	1000
Metsulfuron-methyl	268	292	780
MSMA	1354	5831	79,000
Paraquat dichloride	0	148	< 1
Pendimethalin	0	30,531	3
Permethrin	0	10,000	1
Picloram	1871	0.32	6
Propiconazole	37	3	1
S - Metolachlor	69	7513	5200
Tebuconazole	242	24	58
Terbuthylazine	67	875	830
Terbutryn	100	3056	3100
Triadimenol	1345	0.25	3
Trichlorfon	6	17,647	1100
Trifloxysulfuron sodium	177	100	180
Trifluralin	0	6261	1
Trinexapac-ethyl	0.14	2	< 1

Pesticide active ingredients are not always applied at their maximum permitted rate, so the measure of effect was also calculated based on the minimum and average application rates. This changed the measure of effect values but had little difference on the order of PAIs (Figure S49). The purpose of the PDST is to allow farmers, agronomists, and resellers to select PAIs that pose a lower risk to the aquatic environment, so it is the ranking, rather than the absolute value, that is important. Therefore, the effect of using different application rates on the measure of effect values was tested using a Spearman rank correlation coefficient test to compare the rank of measure of effect values based on maximum, minimum, and average application rates. The Spearman rank correlation coefficient (*r*) was at least 0.95, and the *P* value was < 0.001 for all comparisons.

### Measure of mobility and persistence

*K*_OC_ was selected as a surrogate for mobility in the PDST. Several other pesticide risk indicators have also used *K*_OC_ as an indicator of transport (Kookana et al. 2005; Dabrowski and Balderacchi 2013), while Rice et al. (2010) found that *K*_OC_ correlated reasonably well with experimental pesticide runoff (*R*^2^ = 0.60). Experimental *K*_OC_ values were not available for all PAIs, but there was generally a good relationship between experimental *K*_OC_ values from the PPDB (University of Hertfordshire 2013) and *K*_OC_ values modelled using OPERA (US EPA 2019c) when both were available (Figure S50). The largest difference between experimental and modelled *K*_OC_ values were for diquat dibromide (1917-fold difference) and paraquat dichloride (937-fold difference). Both these PAIs are divalent cations, meaning the OPERA model may not be suitable for such compounds. Consequently, experimental *K*_OC_ values were used where possible and OPERA values were only used when experimental values were not available. Experimental values were available for approximately 66% of the studied PAIs, including diquat dibromide and paraquat dichloride.

While experimental *K*_OC_ correlated reasonably well with modelled *K*_OC_ values and experimental loss data, particularly for the hydrosol (Fig. 1), there are some limitations with using *K*_OC_ as an indicator of mobility. In addition to soil organic carbon, the clay and mineral content of soil is important for sorption for more polar chemicals (Wauchope et al. 2002), while many of the studied PAIs are ionizable compounds (Table S1), so soil pH will also affect sorption for these compounds. As a result, some pesticide risk indicators use PAI pK_a_ to correct for the effect of pH on *K*_OC_ (Kookana and Correll 2008). Pesticide mobility is far more complex than can be captured by one parameter. Pesticide runoff is related to the pesticide soil surface concentration, which can be affected by application rate and dissipation, including leaching into the soil profile (Silburn and Kennedy 2005). Further, some strongly sorbed pesticides, such as paraquat, can be transported in the sediment phase of runoff (Leonard et al. 1979). Despite this, *K*_OC_ was used to determine mobility as *K*_OC_ values are available for a larger number of PAIs compared to other mobility parameters (e.g., experimental or modelled loss data), which is important based on the criteria described in Maud et al. (2001) and in order to be able to include all the PAIs in the PDST.

Many pesticide risk indicators include PAI half-life in soil as a measure of persistence (e.g., Kookana et al. 2005; Le Bellec et al. 2015; Kudsk et al. 2018). This is because while a PAI may have a low sorption capacity, it may decay quickly in the environment, reducing the amount transported to the aquatic environment. It should be noted that pesticide half-life can vary considerably in different tropical Queensland soils and is often different to available soil half-life values in PPDB (Shaw et al. 2013). However, half-life values from PPDB were used as they provide a consistent set of values that allow for direct indicative comparisons between molecules and are readily available.

The measure of mobility and persistence values ranged from approximately 0.01 for PAIs with log *K*_OC_ values greater than 4 (e.g., bifenthrin, diquat dibromide, paraquat dichloride, pendimethalin, permethrin, and trifluralin) to 9977 for the fungicide AI flutriafol. In addition to flutriafol, the PAIs most likely to move from farmland to waterways and persist in the environment were the insecticide AI clothianidin and herbicide AI picloram. There was a 200,000-fold variation in measure of mobility and persistence values for herbicide AIs, a 400,000-fold variation in measure of mobility and persistence values for insecticide AIs, and a 75,000-fold variation in measure of mobility and persistence values for fungicide AIs.

### Aquatic risk

**Fig. 2:**
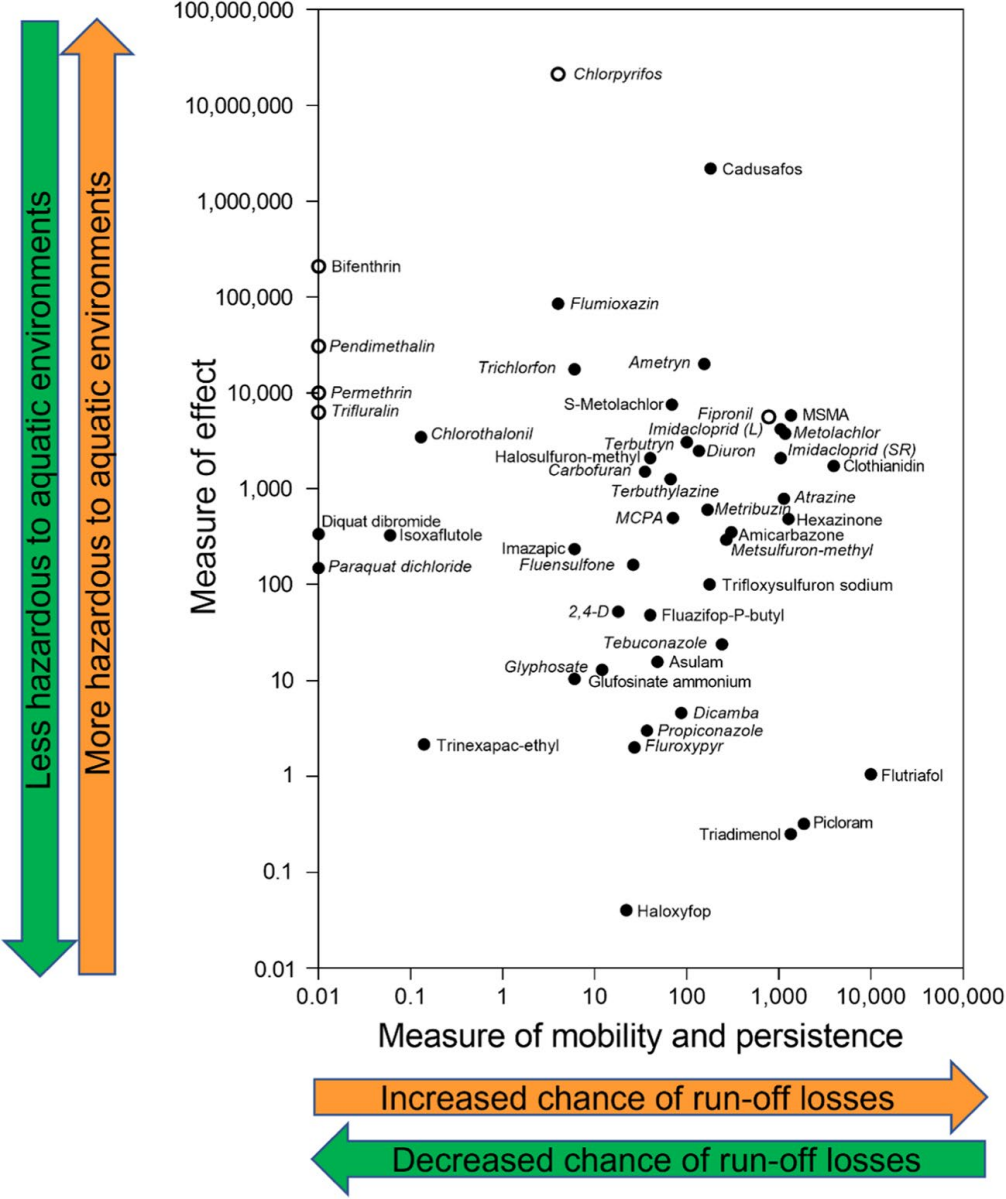
Plot of the measure of effect and the measure of mobility and persistence for all the 47 selected pesticide active ingredient (PAIs) registered for application to sugar cane or its rotation crops. The closed symbols were derived using PC95 values, and the open symbols were derived using PC99 values. PAIs with DGVs or ETVs with moderate, high, or very high reliability are indicated in italics, while PAIs with DGVs or ETVs with unknown, low, or very low reliability are indicated in normal font. Note both slow release (SR) and liquid (L) imidacloprid are included

The measure of effect and measure of mobility and persistence values of the 47 selected PAIs were plotted in Fig. 2. Separate measure of effect and measure of mobility and persistence plots for herbicides, insecticides, and fungicides are provided in Figures S51 to S53. The vertical axis, measure of effect, indicates the potential for a PAI to cause harmful effects should it enter the aquatic environment, with increasing measure of effect values indicating PAIs with a greater potential for environmental harm. The horizontal axis, measure of mobility and persistence, indicates the potential of a PAI to move from farmland into the aquatic environment via runoff and to persist in that environment. Increasing values indicate PAIs with a greater potential to move into and persist in waterways. The aquatic risk is the product of the measure of effect and the measure of mobility and persistence and indicates the likelihood that a PAI will reach a waterway and cause harmful effects. Pesticide active ingredients with larger measure of effect and measure of mobility and persistence values (larger aquatic risk) will occur closer to the top right of Fig. 2. In contrast, PAIs with smaller measure of effect and measure of mobility and persistence values (smaller aquatic risk) will occur closer to the bottom left of Fig. 2. So, if an alternate PAI lies below and to the left of the PAI currently being used, it poses a lower risk to aquatic ecosystems and could be selected for use. Conversely, if an alternate PAI lies above and to the right of the PAI currently being used, then it poses a greater risk to aquatic ecosystems and hopefully would not be chosen. But in many instances, it is not easy to determine if an alternate PAI is less harmful to aquatic ecosystems or not—as it may be below (less toxic) but to the right (more mobile and persistent) of the currently used PAI or it could lie above (more toxic) but to the left (less mobile and persistent) of the currently used PAI. This is where the aquatic risk values (Table 2) become important as they always clearly identify the risk posed by alternate PAIs—the larger the risk value, the greater the risk to aquatic ecosystems and vice versa.

The aquatic risk approach gives equal weighing to measure of effect and measure of mobility and persistence as both determine the likelihood of an adverse effect. For example, if two PAIs, “A” and “B,” have the same measure of effect value, but A is 10 times more mobile and persistent than B, then the risk posed by A will be 10 times greater than for B. Aquatic risk values for the selected PAIs (Table 2) ranged from 0.3 for trinexapac-ethyl to 4.0 × 10^8^ for cadusafos. The insecticide AIs cadusafos and chlorpyrifos posed the greatest potential aquatic risk, followed by the herbicide AI MSMA. Fungicides often had lower measure of mobility and persistence and lower measure of effect values than herbicides and insecticides, resulting in lower aquatic risk values. While not focusing on sugar cane and using different methodologies, the PAI aquatic risk rankings from the literature overlapped to some extent with the current study. For example, PAIs chlorpyrifos, clothianidin, and atrazine were among the top priority PAIs in Canadian waters (Anderson et al. 2021), while metolachlor, imidacloprid, and fipronil were among the prioritized compounds of concern in the Great Lakes Basin (Oliver et al. 2023).

**Fig. 3:**
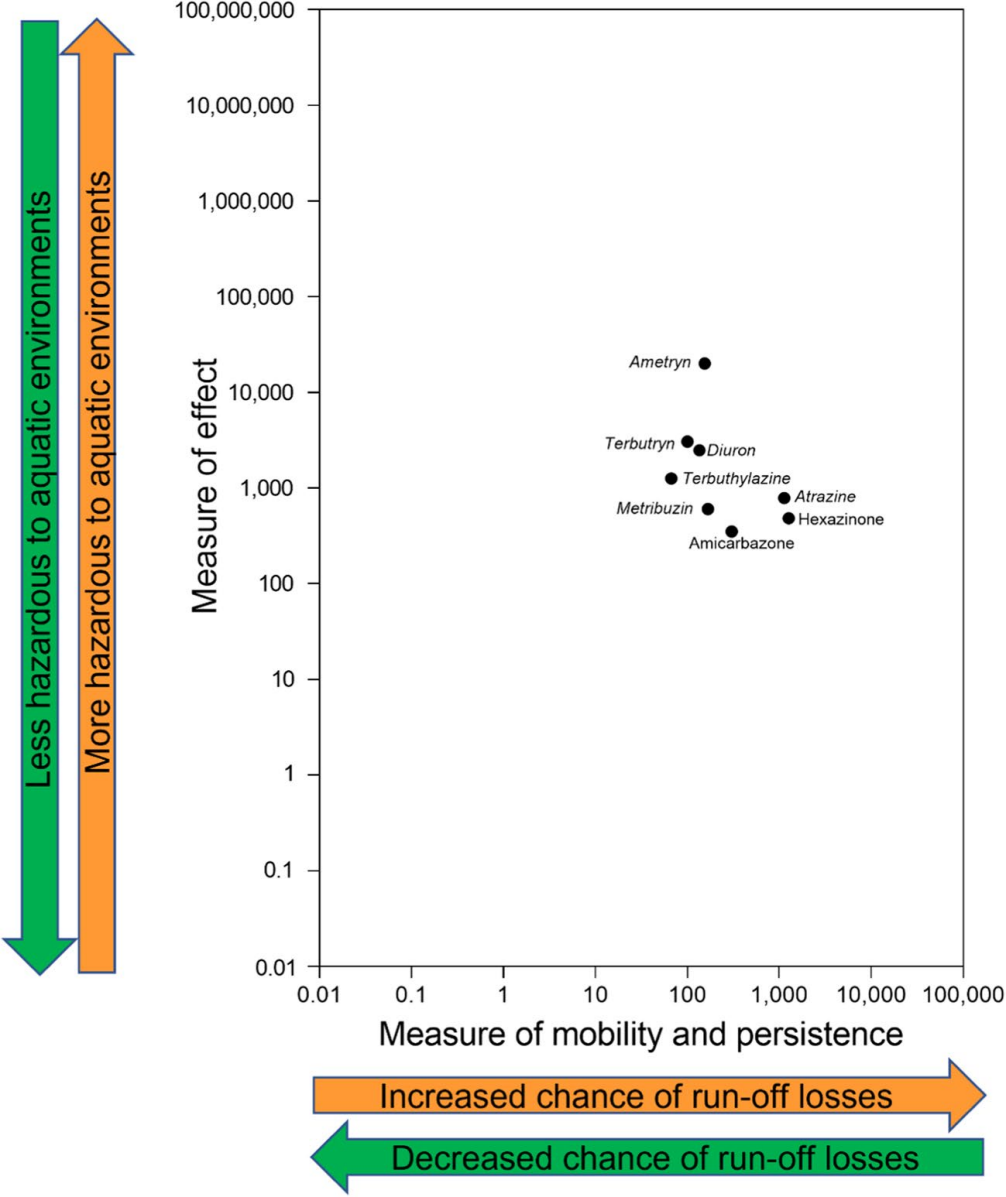
Plot of the measure of effect and the measure of mobility and persistence for PSII herbicide AIs registered for application to sugar cane. Pesticide active ingredients (PAIs) with DGVs or ETVs with moderate, high, or very high reliability are indicated in italics, while PAIs with DGVs or ETVs with unknown, very low, or low reliability are indicated in normal font

Focusing on the example of PSII herbicide AIs, ametryn poses the highest aquatic risk, followed by atrazine, hexazinone, diuron, terbutryn, amicarbazone, metribuzin, and terbuthylazine (Fig. 3, Table 2). Ametryn has the highest measure of effect (20,000) of any PSII herbicide AI and the fifth highest measure of mobility and persistence (155) resulting in an aquatic risk of 3.1 × 10^6^ (Table 2). Conversely, hexazinone has the second lowest measure of effect (480), but the highest measure of mobility and persistence of any studied PSII herbicide (1274), resulting in the third highest aquatic risk (6.1 × 10^5^).

### Comparison with other pesticide risk indicators

Compared to other pesticide risk indicators, the PDST does not include site-specific information, such as soil type, rainfall, or slope. As such, the PDST should be viewed as a generic tool to select PAIs that pose a lower aquatic risk based on toxicity, application rate, mobility, and persistence. However, factors such as land slope or proximity to sensitive sites or waterbodies can be considered by the PDST in a qualitative manner by selecting less mobile and persistent PAIs. Overall, the preference should be to select the least mobile and persistent and least toxic PAIs—PAIs with the lowest aquatic risk. It is particularly important to select less mobile PAIs in situations where the ground slope is high, or the point of application is near a sensitive receptor such as a waterway. It is also important to note that the PDST only considers PAI transport to surface water via surface runoff and does not consider loss due to leaching (although it may be appropriate for this purpose as the characteristics considered in the PDST would also be relevant to leaching), soil erosion, or spray drift. Other more advanced models could be applied in parallel to the PDST if more site-specific guidance is required.

Of the available risk indicators, the pesticide impact rating index (PIRI) was used to rank the risk of 25 herbicide AIs used on sugar cane, including six PSII herbicide AIs (ametryn, atrazine, diuron, hexazinone, terbutryn, and terbuthylazine) in different GBR regions (Davis et al. 2014). PIRI determines a risk class separately for algae, daphnids (*Daphnia* sp.), and fish (rainbow trout) using the single lowest acute toxicity value for each group of organisms. The lowest toxicity value is not modified using an assessment factor, nor was a species sensitivity distribution used. The outputs of PIRI for the six PSII herbicides, which were based on mobility in a Wet Tropics silt soil and toxicity to algae, were compared to the outputs from the PDST (Table 3). Risk classes based on toxicity to algae were selected as algae are the most sensitive group of organisms to PSII herbicides. While it is difficult to compare the aquatic risk values and ranking from the PDST with the risk classes from PIRI, it is clear that the ranking of the PSII herbicide AIs from greatest risk to lowest risk is different. Irrespective of this, the main point of this comparison is to show that grouping PAIs into various risk classes decreases the ability to determine if one PAI is less harmful than another in the same risk class. For example, it is not possible to determine whether atrazine or metribuzin, which are both considered high to very high risk, would be less harmful based on the PIRI risk classes (Table 3). However, 3.6 times more atrazine is needed to provide sufficient residual control of broadleaf weeds compared to metribuzin (e.g., 0.75 kg PAI/ha of metribuzin vs 2.7 kg PAI/ha of atrazine); thus, including these two PAIs in the same risk class is misleading as atrazine will pose a greater risk to the aquatic environment. The aquatic risk values generated by the PDST (Table 3) permit users to determine which of these PAIs would be less harmful for aquatic ecosystems. This inability to differentiate the risk of PAIs in the same risk class could lead to PAIs that are worse for aquatic environments being selected, as outlined in the example above.

**Table 3:** The ranking, from highest to lowest risk, of six photosystem II inhibiting (PSII) herbicides by the PIRI (Davis et al. 2014) and PDST methods. The PIRI classes are based on mobility and toxicity to algae on a Wet Tropics silt soil

PIRI ranking and risk class	PDST ranking and aquatic risk values (× 100)
Ametryn, diuron, terbutryn (very high risk)	Ametryn (31,000)
Atrazine, metribuzin (high to very high risk)	Atrazine (8900)
Hexazinone (high risk)	Hexazinone (6100)
	Diuron (3400)
	Terbutryn (3100)
	Metribuzin (1000)

As an example, the cost per hectare of the three PSII herbicide AIs in the very high risk class (based on PIRI) at the top rate for application to sugar cane is $37.87 for diuron 900 WG (1.9 kg/ha), $78.77 for ametryn 800 WG (2.8 kg/ha), and $87.47 for terbutryn (as terbutryn + MCPA at 4 L/ha) (based on costs obtained from a commercial pesticide supplier in March 2023). Assuming the three PAIs have the same efficacy, it is quite likely that diuron would be selected due to it posing the same risk but being roughly half the cost. However, the continuous aquatic risk values provide by the PDST combined with the cost could lead to a different decision being made. This demonstrates the advantage of presenting the aquatic risk as a continuum of values, rather than risk classes, as it provides clearer advice to users.

Another recent study used experimental pesticide runoff concentrations and toxicity relative to diuron to rank the risk of 12 herbicides applied to sugar cane (Silburn et al. 2023). Runoff was assessed using simulated rainfall for both bare soil and soil with green cane trash blanket for four locations in the Great Barrier Reef catchment area. Similar to the current study, PSII herbicides ametryn and hexazinone were ranked as having a greater risk, while pendimethalin, glyphosate, and fluroxypyr had a lower risk ranking.

Compared to some other pesticide risk indicators, the PDST meets all the criteria proposed by Maud et al. (2001) as being important in a pesticide risk indicator. The PDST uses readily available data (i.e., PAI application rate, *K*_OC_, half-life, and DGV or ETV), is simple and transparent, excludes risks to humans, and does not use a weighting system to rank pesticide risk. The PDST was also developed through extensive consultation with stakeholders to ensure it was fit for purpose. Further, PDST generates a single aquatic risk value based on a species sensitivity distribution that used as much of the available chronic toxicity data as possible. The aquatic risk value allows differentiation between the different PAIs, with aquatic risk calculated based on the likelihood that a PAI will reach a waterway and cause harmful effects. Given the generic nature of the PDST, relatively small differences in aquatic risk values should be interpreted cautiously whereas large differences are likely to indicate actual differences in risk under field conditions. For example, dicamba, picloram and 2,4-D, which are all synthetic auxins, have aquatic risk values 400, 600, and 900, respectively, and therefore may pose similar risks under field conditions. In contrast, the PSII herbicides atrazine and amicarbazone have aquatic risk values of approximately 900,000 and 110,000 respectively. Amicarbazone is likely to pose a lower risk under field conditions and be a better option.

Risk classes were not used in the PDST as the cutoff points for each class will always be arbitrary, while methods that treat risk as a discrete entity do not allow differentiation between PAIs within the same risk class.

### Application and future work

The measure of effect and aquatic risk values presented in Fig. 2 and Table 2 were calculated assuming the maximum application rate and a broadcast spray regime, but farmers may use different application rates or spray regimes (e.g., spot or band spraying). Therefore, an interactive Excel version of the PDST was developed to allow users to enter the PAI concentration percentage and product application rate, as well as the percentage of land to be spot or band sprayed (see the ESM). For example, the aquatic risk of a diuron decreases 10-fold from 3370 to 339 in moving from broadcast application to band spraying of 10% of the field (assuming band spraying applies 10% of the maximum permitted product application rate for broadcast spraying). By considering application rates, the efficacy of different PAIs is included in the PDST and thus comparisons of the potential aquatic risk pesticide PAIs pose are done on an equal (or near equal) efficacy basis. Further, the interactive Excel version of the PDST allows users to compare the aquatic risk of chemical groups (e.g., organophosphates or PSII inhibitors) to help select less harmful PAIs that control the same pest issue as more harmful PAIs.

The interactive Excel version of the PDST can also be used to compare the aquatic risk of current and alternative tank mixes, where combinations of PAIs are mixed for a single application. Users can sum the aquatic risk values for the PAIs used in each tank mix and then compare the results. By summing the aquatic risk, it is assumed that the PAIs have the same mode of action (e.g., exert their toxicity in the same manner) when, in reality, the PAIs in a tank mix may have different modes of action. When pesticides with different modes of action are combined, their joint toxicity is typically described by the independent action (IA) model which usually leads to lower estimates of joint toxicity than the concentration addition (CA) model (Faust et al. 1994; Backhaus et al. 2000; Dyer et al. 2000; Chèvre et al. 2006; Junghans et al. 2006); however, the toxicity values are generally not statistically significantly lower (Dyer et al. 2011). Mixes of pesticides with different modes of action can also have antagonistic (where the joint toxicity is less than predicted by the CA and IA models) or synergistic (where the joint toxicity is more than predicted by the CA and IA models) joint toxicity. Other than conducting toxicity tests, there is currently no way of predicting whether a mixture will have synergistic or antagonistic joint toxicity (De Zwart and Posthuma 2005). However, a number of meta-studies have examined the available mixture toxicity data and have come to the conclusion that the vast majority of mixtures conform to either the CA or IA models of joint toxicity (Belden et al. 2007; Dyer et al. 2011; Warne et al. 2020). Therefore, the summed aquatic risk for tank mixes should be considered as an indicative aquatic risk, not a definitive aquatic risk, as we do not have information about the combined toxicity of each tank mix.

When applying the PDST, it is still necessary to consider the general principles related to pesticide application and integrated pest management, such as varying the pesticides used to minimize developing pest resistance and spraying for pest control prior to the start of the wet season, where possible. The PDST does not override these principles but should be considered in conjunction with them. For example, the PDST can be used to select several lower aquatic risk pesticide PAIs for rotation. Further, the PDST is based on PAIs, but pesticide formulations can also contain adjuvants and wetting agents, which have not been considered in the PDST.

The PDST can also provide guidance on pesticide selection in different seasons. The regions where sugar cane is grown in Queensland have a distinct wet and dry season. The wet season, which typically runs from November to April, is when most of the rain occurs. During the wet season, there is a far greater chance that pesticides will be transported to rivers and creeks, and it is therefore increasingly important to select PAIs that have low measure of mobility and persistence values (further to the left in Fig. 2). During the dry season, when the chance of pesticides being transported to rivers and creeks is dramatically lower, the mobility and persistence of the PAIs is not such an important issue and choosing a PAI with lower measure of effect (lower in Fig. 2) might be more important to consider when selecting PAIs for use.

As part of general principles of pesticide management, pesticide use should always be minimized, for example, by only applying them where they are actually required rather than using them in a prophylactic manner or applying them as a form of insurance. It should be noted that a prophylactic application does not include those applications that are conducted as a well-considered preventative measure. In many situations, pest occurrence is known to occur in conjunction with particular environmental triggers, so products that work to prevent the pest from occurring or proliferating can be used to strategically mitigate such issues. An example is the use of pre-emergent herbicides, which can prevent or inhibit the germination of target weeds before they emerge from the ground. These products are a very important component of an integrated management system.

The PDST is designed to assist farmers, agronomists, extension officers, and resellers select PAIs that pose a lower risk to the aquatic environment than the PAIs that they currently use to control a particular pest. The PDST only provides information on the potential aquatic risk that PAIs pose. It does not include other important factors that are likely to be considered in deciding which PAIs are to be applied to sugar cane, such as the PAI efficacy against the target pest or its cost per hectare. The PDST only considers PAIs that are registered for use by the sugar cane industry, and therefore, it does not identify PAIs that cannot be used. Also, as it provides generic information, it should be used in combination with advice from resellers, agronomists, or extension officers about the most appropriate pesticide to use in a given situation (i.e., weed or crop types, integrated management strategies, economics, efficacy, site characteristics). The PDST can be used as a component of pesticide management planning, as is being done in Project Bluewater in the Mackay Whitsunday and Burdekin regions of Queensland, Australia, which provides participating sugar cane farmers with pesticide management plans for each parcel of land on a farm.

The current version of the PDST had been extensively road-tested for its appropriateness and ease of use and interpretation with farmers, resellers, and agronomists, as well as agricultural and ecotoxicological scientists. All groups appreciated the amount and robustness of the data that underpins the PDST and that the PDST summarized and presented the results of the assessment in a simple graphical format and with numerical aquatic risk values that were easy to understand and increased their understanding of the key factors controlling the potential risk posed by PAIs. Initially, it was felt that the generic PDST would be followed by the development of an app that could give guidance on pesticide use at an individual paddock scale. There was strong support for this among stakeholders initially. However, once the PDST was developed and presented to stakeholders, they were asked whether they would prefer the development of an app for the 47 selected PAIs or for the PDST to be expanded to cover all the additional PAIs that are currently registered for use on mung bean, soybean, corn, and rice—the four main crops grown in rotation with sugar cane in Queensland. Stakeholders overwhelmingly supported the expansion of the PDST to cover all the PAIs used in sugar cane farming. This will therefore be the focus of future work. In addition, ongoing work is required to evaluate the success of the PDST in changing pesticide use. This will be achieved through ongoing monitoring of pesticides in rivers in the GBR catchments by the Great Barrier Reef Catchment Loads Monitoring Program conducted by the Queensland Department of Environment and Science and pesticide application data provided by participating farmers in Project Bluewater. This will be the subject of future publications. Further, while this study focuses on PAIs applied to sugar cane in Queensland, the generic nature of the PDST means that it can be applied internationally and to any PAIs with sufficient toxicity, mobility, and persistence data.

Currently, training and educational materials are being developed to inform stakeholders and facilitate adoption of the PDST.

## Conclusions

The current study developed a simple Pesticide Decision Support Tool (PDST) to assess the potential aquatic risk of active ingredients in pesticides that are registered for application to sugar cane and associated rotation crops in Queensland, Australia. This considered the likelihood that a PAI will reach a waterway and cause an adverse effect. The PDST was developed in collaboration with representatives of government, pesticide users, advisers, and resellers. It considers application rate, and thus, comparisons of potential aquatic risk are made on an equal (or near equal) efficacy basis. Insecticide AIs, such as cadusafos, chlorpyrifos, and clothianidin, posed the greatest aquatic risk, followed by herbicide AIs MSMA, metolachlor, and ametryn. Fungicide AIs typically posed lower aquatic risk. Compared to other pesticide indicators that use risk classes, the PDST presents the aquatic risk as a continuum of values, allowing users to easily identify and select less environmentally harmful PAIs to control the pest issue they face, should they wish to. Further, the PDST is available as an interactive Excel tool, which allows farmers to calculate the aquatic risk based on the spray regimes they use or compare different tank mixes. The PDST does not include site-specific information, such as soil type, rainfall, or slope, and consequently should be viewed as a generic tool to select PAIs that pose a lower potential aquatic risk. The PDST should also be applied in conjunction with advice from agronomists and extension officers. Further work is required to expand the PDST to all PAIs used in crops grown in rotation with sugar cane.

### Supplementary information

ESM 1

ESM 2

ESM 3

ESM 4

## Data Availability

All data used in this study are available in the ESM or from the corresponding author on request.
