# Supplementary material for: A Pesticide Decision Support Tool to guide the selection of less environmentally harmful pesticides for the sugar cane industry
Source: Environ Sci Pollut Res Int. 2023 Sep 25;30(49):108036–50. doi: 10.1007/s11356-023-29814-w (PMC10611884; doi:10.1007/s11356-023-29814-w)
Supplement: Supplementary file 1 — ESM 1 [file 11356_2023_29814_MOESM1_ESM.docx]

**Electronic Supplementary Material**: A Pesticide Decision Support Tool to Guide the Selection of Less Environmentally Harmful Pesticides for the Sugar Cane Industry

Michael St. John Warne^1,2,3*^, Peta A. Neale^1^ and Michael J. Macpherson^4^

^1^School of Earth and Environmental Sciences, University of Queensland, Brisbane, Queensland 4108, Australia.

^2^Water Quality and Investigations, Environmental Monitoring and Assessment Science, Science Delivery, Department of Environment and Science, Brisbane, Queensland 4102, Australia.

^3^Centre for Agroecology, Water and Resilience, Coventry University, Coventry, United Kingdom.

^4^Farmacist Pty Ltd, Mackay, Queensland

Email: michael.warne@uq.edu.au

Table S1: Physicochemical properties and chemical abstract number (CAS No.) of the 47 studied pesticide active ingredients.

| **Name** | **CAS No.** | **Molecular weight (g/mol)** | **Log K_OW_^a^** | **Solubility (mg/L, 20°C)^b^** | **K_OC_ (L/kg)** | **Soil** t_1/2_ **(days) _b, d_** | **Water** t_1/2_  **(days)** | **pK_a_‡** | **% of compound in neutral form at pH 7** |
| --- | --- | --- | --- | --- | --- | --- | --- | --- | --- |
| 2,4-D | 94-75-7 | 221.03 | 2.81 | 24,300 | 39^b^ | 4.4 | 7.7^e^ | 3.4 (acid)^b^ | 0.03% |
| Ametryn | 834-12-8 | 227.33 | 2.98 | 200 | 316^b^ | 37 | 30^e^ | 4.1 (base)^d^ | 99.87% |
| Amicarbazone | 129909-90-6 | 241.30 | 1.90 | 4,600 | 30^b^ | 21 | 60^f^ | no pK_a_ | 100% |
| Asulam | 3337-71-1 | 230.24 | -0.27 | 962,000 | 33^c^ | 3.2 | 63^g^ | 1.3 (acid)^b^ | 0.00% |
| Atrazine | 1912-24-9 | 215.69 | 2.61 | 35 | 100^b^ | 75 | 80^h^ | 1.7 (base)^b^ | 100% |
| Bifenthrin | 82657-04-3 | 422.87 | 6.79^c^ | 0.001 | 236,610^b^ | 102 | 8^e^ | no pK_a_ | 100% |
| Cadusafos | 95465-99-9 | 270.39 | 3.90 | 245 | 396^c^ | 38 | 37^e^ | no pK_a_ | 100% |
| Carbofuran | 1563-66-2 | 221.26 | 2.32 | 322 | 87c | 29 | 6.1^e^ | no pK_a_ | 100% |
| Chlorothalonil | 1897-45-6 | 265.91 | 3.05 | 0.81 | 2,632^b^ | 3.5 | 0.82^e^ | no pK_a_ | 100% |
| Chlorpyrifos | 2921-88-2 | 350.57 | 4.96 | 1.05 | 5,509^b^ | 386 | 5^e^ | no pK_a_ | 100% |
| Clothianidin | 210880-92-5 | 249.67 | 0.70 | 340 | 123^b^ | 545 | 40^e^ | 11.1 (base)^b^ | 0.01% |
| Dicamba | 1918-00-9 | 221.03 | 2.21 | 250,000 | 44^c^ | 9.6 | 40^e^ | 1.87 (acid)^b^ | 0.00% |
| Diquat dibromide | 85-00-7 | 344.05 | 1.05 | 718,000 | 2,185,000^b^ | 2345 | 1^e^ | cation† | - |
| Diuron | 330-54-1 | 233.09 | 2.68 | 35.6 | 680^b^ | 146 | 8.8^e^ | no pK_a_ | 100% |
| Fipronil | 120068-37-3 | 437.14 | 4.00 | 3.78 | 754^c^ | 142 | 54^e^ | no pK_a_ | 100% |
| Fluazifop-P-butyl | 79241-46-6 | 383.37 | 4.50 | 0.93 | 3,394^b^ | 25 | 45^e^ | no pK_a_ | 100% |
| Fluensulfone | 318290-98-1 | 291.69 | 3.10^c^ | 465^c^ | 459^c^ | 10 | 21^f^ | no pK_a_ | 100% |
| Flumioxazin | 103361-09-7 | 354.34 | 2.55 | 0.786 | 889^b^ | 21.9 | 2^e^ | no pK_a_ | 100% |
| Fluroxypyr | 69377-81-7 | 255.03 | 2.02 | 6,500 | 107^c^ | 13.1 | 11^e^ | 2.9 (acid)^b^ | 0.01% |
| Flutriafol | 76674-21-0 | 301.30 | 2.29 | 95 | 72^c^ | 1358 | 36^e^ | 2.3 (acid)^b^ | 0.00% |
| Glufosinate ammonium | 77182-82-2 | 198.16 | -1.17 | 500,000 | 600^b^ | 7.4 | 7.2^e^ | 1.9 (acid)^i^ | 0.00% |
| Glyphosate | 1071-83-6 | 169.07 | -3.40 | 10,500 | 1,424^b^ | 16.1 | 9.9^e^ | 2.34 (acid)^b^ | 0.00% |
| Halosulfuron-methyl | 100784-20-1 | 434.81 | -0.02 | 10.2 | 109^b^ | 26.7 | 8.1^e^ | 3.44 (acid)^b^ | 0.03% |
| Haloxyfop | 69806-34-4 | 361.70 | 3.78 | 1.6 | 75^b^ | 9 | 12^f^ | 2.9 (acid)^b^ | 0.01% |
| Hexazinone | 51235-04-2 | 252.32 | 1.85 | 33,000 | 54^b^ | 105 | 56^f^ | 2.2 (base)^b^ | 100% |
| Imazapic | 104098-48-8 | 275.31 | 2.47 | 2,230 | 137^b^ | 120 | 0.3^f^ | 2.0 (acid)^b^ | 0.00% |
| Imidacloprid | 138261-41-3 | 255.66 | 0.57 | 610 | 118^c^ | 191 | 30^e^ | no pK_a_ | 100%* |
| Isoxaflutole | 141112-29-0 | 359.32 | 2.32 | 6.2 | 145^b^ | 0.9 | 0.36^e^ | no pK_a_ | 100% |
| MCPA | 94-74-6 | 200.62 | 3.25 | 29,390 | 51^c^ | 24 | 13.5^e^ | 3.7 (acid)^b^ | 0.05% |
| Metolachlor | 51218-45-2 | 283.80 | 3.13 | 530 | 261^c^ | 90 | 88^e^ | no pK_a_ | 100% |
| Metribuzin | 21087-64-9 | 214.29 | 1.70 | 10,700 | 55^c^ | 7 | 41^e^ | 2.5 (base)^i^ | 100% |
| Metsulfuron-methyl | 74223-64-6 | 381.36 | 2.20 | 2,790 | 37^c^ | 10 | 115^e^ | 3.8 (acid)^b^ | 0.06% |
| MSMA | 2163-80-6 | 161.95 | -3.10 | 580,000 | 44^c^ | 200 | 30^e^ | 4.2 (acid)^i^ | 0.16% |
| Paraquat dichloride | 1910-42-5 | 257.16 | -4.22 | 620,000 | 1,000,000^b^ | 3000 | 10^e^ | cation† | - |
| Pendimethalin | 40487-42-1 | 281.31 | 5.18 | 0.33 | 17,491^b^ | 182 | 4^e^ | 2.8 (acid)^b^ | 0.01% |
| Permethrin | 52645-53-1 | 391.29 | 6.50 | 0.2 | 100,000^b^ | 13 | 23^e^ | no pK_a_ | 100% |
| Picloram | 1918-02-1 | 241.45 | 1.90 | 560 | 13^b^ | 82.8 | 81^e^ | 2.3 (acid)^b^ | 0.00% |
| Propiconazole | 60207-90-1 | 342.22 | 3.72 | 150 | 1,086^b^ | 71.8 | 6^e^ | 1.1 (base)^b^ | 100% |
| S-Metolachlor | 87392-12-9 | 283.80 | 3.13 | 480 | 261^c^ | 51.8 | 9^e^ | no pK_a_ | 100% |
| Tebuconazole | 107534-96-3 | 307.82 | 3.70 | 36 | 994^c^ | 63 | 43^e^ | 5.0 (base)^b^ | 99.01% |
| Terbuthylazine | 5915-41-3 | 229.71 | 3.21 | 6.6 | 226 ^c^ | 72 | 6^e^ | 1.9 (base)^b^ | 100% |
| Terbutryn | 886-50-0 | 241.36 | 3.74 | 25 | 2,432^b^ | 74 | 27^e^ | 4.3 (base)^b^ | 99.80% |
| Triadimenol | 55219-65-3 | 295.77 | 3.08 | 72 | 750^b^ | 250 | 53^e^ | no pK_a_ | 100% |
| Trichlorfon | 52-68-6 | 257.43 | 0.51 | 120,000 | 10^b^ | 18 | 1.2^e^ | no pK_a_ | 100% |
| Trifloxysulfuron sodium | 199119-58-9 | 459.33 | 1.32 | 25,700 | 306^b^ | 70 | 18^f^ | 4.8 (acid)^b^ | 0.57% |
| Trifluralin | 1582-09-8 | 335.28 | 5.28 | 0.221 | 15,800^b^ | 134 | 13^e^ | no pK_a_ | 100% |
| Trinexapac-ethyl | 95266-40-3 | 252.27 | 1.60 | 10,200 | 74^c^ | 0.16 | 4.2^e^ | 4.6 (acid)^b^ | 0.37% |

^a^US EPA EpiSuite (US EPA 2012); ^b^PPDB (University of Hertfordshire 2013); ^c^US EPA OPERA (US EPA 2019a); ^d^PubChem database (National Center for Biotechnology Information 2019); ^e^water phase T_1/2_ obtained from ^b^; ^f^aqueous photolysis T_1/2_ obtained from ^b^; ^g^aqueous hydrolysis T_1/2_ from ^b, d^; ^h^water -sediment T_1/2_ from ^b^; ^i^ChemAxon (ChemAxon, 2019).

†Paraquat and diquat are double positively charged cations (National Center for Biotechnology Information 2019).
‡Some active ingredient do not contain ionizable functional groups, so no pK_a_ could be calculated.

*Zwitterion at pH 7 (i.e. contains positively and negatively charged functional groups but has a net neutral charge).

Table S2: The forty-seven active ingredients (AI), their proportion in commercial products and the maximum, minimum and average application rates for both the product and AI based on pesticide labels from PubCRIS (APVMA 2019).

| **Active ingredient (AI)** | **Active ingredient concentration (%)** | **Product Application Rate** | | | **Active Ingredient Application Rate** | | |
| --- | --- | --- | --- | --- | --- | --- | --- |
|  |  | *Maximum*  *application rate (kg product/ha)* | *Minimum*  *application rate (kg product/ha)* | *Average application rate (kg product /ha)* | *Maximum*  *application rate  (kg AI/ha)* | *Minimum*  *application rate (kg AI/ha)* | *Average*  *application rate  (kg AI/ha)* |
| 2,4-D | 62.5 | 3.50 | 1.80 | 2.65 | 2.19 | 1.13 | 1.66 |
| Ametryn | 25 | 8.00 | 6.00 | 7.00 | 2.00 | 1.50 | 1.75 |
| Amicarbazone | 70 | 1.00 | 0.50 | 0.75 | 0.70 | 0.35 | 0.53 |
| Asulam | 40 | 8.50 | 8.50 | 8.50 | 3.40 | 3.40 | 3.40 |
| Atrazine | 90 | 3.30 | 2.20 | 2.75 | 2.97 | 1.98 | 2.48 |
| Bifenthrin | 25 | 0.15 | 0.15 | 0.15 | 0.04 | 0.04 | 0.04 |
| Cadusafos | 10 | 40.0 | 20.0 | 30.0 | 4.00 | 2.00 | 3.00 |
| Carbofuran | 10 | 30.0 | 30.0 | 30.0 | 3.00 | 3.00 | 3.00 |
| Chlorothalonil^a^ | 72 | 2.30 | 1.80 | 2.05 | 1.66 | 1.30 | 1.48 |
| Chlorpyrifos | 50 | 2.00 | 0.35 | 1.17 | 1.00 | 0.18 | 0.59 |
| Clothianidin | 20 | 2.50 | 1.25 | 1.88 | 0.50 | 0.25 | 0.38 |
| Dicamba | 50 | 0.56 | 0.28 | 0.42 | 0.28 | 0.14 | 0.21 |
| Diquat dibromide | 11.5 | 3.20 | 1.20 | 2.20 | 0.37 | 0.14 | 0.25 |
| Diuron | 90 | 1.90 | 0.28 | 1.02 | 1.71 | 0.25 | 0.91 |
| Fipronil | 20 | 0.25 | 0.25 | 0.25 | 0.05 | 0.05 | 0.05 |
| Fluazifop-P-butyl^b^ | 12.8 | 1.65 | 0.82 | 1.24 | 0.21 | 0.10 | 0.16 |
| Fluensulfone | 48 | 4.00 | 4.00 | 4.00 | 1.92 | 1.92 | 1.92 |
| Flumioxazin | 50 | 0.70 | 0.09 | 0.40 | 0.35 | 0.05 | 0.20 |
| Fluroxypyr | 40 | 1.50 | 0.65 | 1.08 | 0.60 | 0.26 | 0.43 |
| Flutriafol | 50 | 0.50 | 0.50 | 0.50 | 0.25 | 0.25 | 0.25 |
| Glufosinate ammonium | 20 | 5.00 | 1.00 | 3.00 | 1.00 | 0.20 | 0.60 |
| Glyphosate | 54 | 6.00 | 2.70 | 4.35 | 3.24 | 1.46 | 2.35 |
| Halosulfuron - methyl | 75 | 0.13 | 0.07 | 0.10 | 0.10 | 0.05 | 0.07 |
| Haloxyfop^b^ | 52 | 0.15 | 0.10 | 0.13 | 0.08 | 0.05 | 0.07 |
| Hexazinone | 13.2 | 4.00 | 0.53 | 2.27 | 0.53 | 0.07 | 0.30 |
| Imazapic | 24 | 0.40 | 0.30 | 0.35 | 0.10 | 0.07 | 0.08 |
| Imidacloprid (Liquid) | 35 | 1.44 | 0.72 | 1.05 | 0.50 | 0.25 | 0.37 |
| Imidacloprid (Slow release)^c^ | 5 | 3.75 | 2.50 | 3.00 | 0.19 | 0.13 | 0.16 |
| Isoxaflutole | 75 | 0.20 | 0.10 | 0.15 | 0.15 | 0.08 | 0.11 |
| MCPA | 75 | 1.45 | 0.93 | 1.19 | 1.09 | 0.70 | 0.89 |
| Metolachlor | 96 | 1.80 | 1.10 | 1.45 | 1.73 | 1.06 | 1.39 |
| Metribuzin | 75 | 2.00 | 0.64 | 1.32 | 1.50 | 0.48 | 0.99 |
| Metsulfuron-methyl^d^ | 60 | 0.012 | 0.0083 | 0.010 | 0.007 | 0.005 | 0.006 |
| MSMA | 72 | 6.60 | 6.60 | 6.60 | 4.75 | 4.75 | 4.75 |
| Paraquat dichloride | 25 | 1.60 | 1.20 | 1.40 | 0.40 | 0.30 | 0.35 |
| Pendimethalin | 44 | 3.40 | 2.25 | 2.83 | 1.50 | 0.99 | 1.24 |
| Permethrin | 50 | 0.20 | 0.10 | 0.15 | 0.10 | 0.05 | 0.08 |
| Picloram | 7.5 | 2.40 | 0.70 | 1.55 | 0.18 | 0.05 | 0.12 |
| Propiconazole^e^ | 25 | 0.12 | 0.06 | 0.09 | 0.03 | 0.02 | 0.02 |
| S - Metolachlor | 96 | 1.80 | 1.10 | 1.45 | 1.73 | 1.06 | 1.39 |
| Tebuconazole | 75 | 0.17 | 0.17 | 0.17 | 0.12 | 0.12 | 0.12 |
| Terbuthylazine^b^ | 87.5 | 1.20 | 0.86 | 1.03 | 1.05 | 0.75 | 0.90 |
| Terbutryn | 27.5 | 4.00 | 2.00 | 3.00 | 1.10 | 0.55 | 0.83 |
| Triadimenol^e^ | 25 | 0.12 | 0.06 | 0.09 | 0.03 | 0.02 | 0.02 |
| Trichlorfon | 50 | 1.20 | 1.20 | 1.20 | 0.60 | 0.60 | 0.60 |
| Trifloxysulfuron sodium | 1.85 | 2.00 | 1.50 | 1.75 | 0.04 | 0.03 | 0.03 |
| Trifluralin | 48 | 3.00 | 2.30 | 2.65 | 1.44 | 1.10 | 1.27 |
| Trinexapac-ethyl | 25 | 0.80 | 0.80 | 0.80 | 0.20 | 0.20 | 0.20 |

^a^ application rates based on sweet corn; ^b^ application rates based on soybeans; ^c^ Slow release imidacloprid in pellet form is applied every four years, so the application rate was divided by 4; ^d^ application rate of 5 to 7 g AI/ha (Allan Blair, pers. Comm.); ^e^ 20 mL/100 L applied, with 300 L/ha considered a typical application rate and 600 L/ha considered a high rate (Rob Sluggett, pers. comm.)

Figure S1: Plot of the rank of experimental pesticide active ingredient runoff loss in well-drained deep sandy soil and poorly drained hydrosol and rank of K_OC_ values. NB: MCPA not included in bottom left graph.


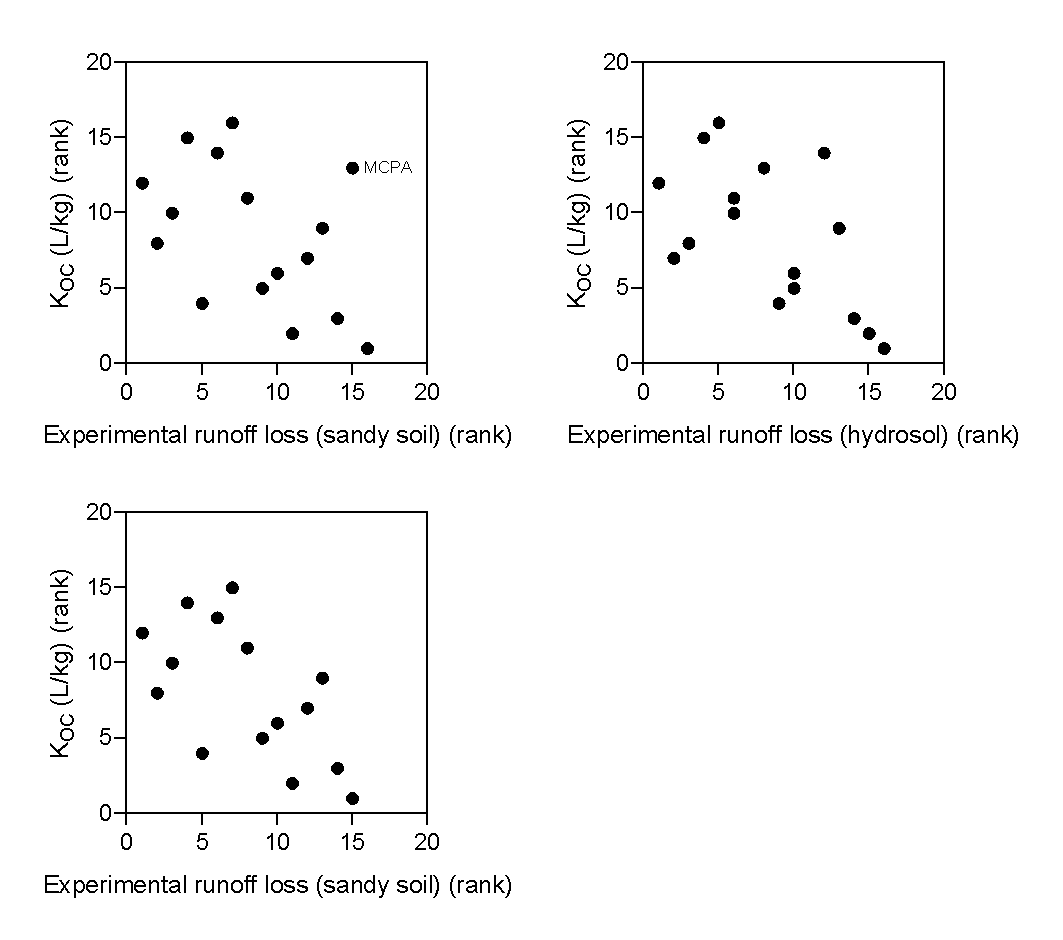


**Section S1: Derivation of ecotoxicity threshold values**

Ecotoxicity threshold values (ETVs) were derived for active ingredients for which there were not current (ANZECC and ARMCANZ 2000) or proposed Default Guideline Values (DGVs) (King et al. 2017a; King et al. 2017b). The ETVs were calculated using the nationally endorsed methods of Warne et al. (2018). Only freshwater species were considered when deriving the ETVs.

### 2,4-D in freshwater

2,4-D (2,4-dichlorophenoxyacetic acid, CAS No. 94-75-7) is a selective, systemic phenoxy herbicide, or more specifically a phenoxyacetic herbicide. It mimics the plant hormone, auxin, and exerts its toxicity by acidifying the cell walls of plants, which causes cells to elongate in an uncontrolled and disorganised manner, ultimately leading to plant death (Walters 1999). 2,4-D also affects the metabolism of plants by affecting enzyme activity, respiration and cell division (Walters 1999). The sensitivity of all organism types for which there was 2,4-D ecotoxicity data was analysed using the bimodality coefficient (BC) (Warne et al. 2018). This indicated the data were not bimodal (BC= 0.433 while the minimum value indicating a bimodal distribution is 0.55). A box and whisker plot (Figure S2) of the 2,4-D data was generated using phototroph and heterotroph ecotoxicity data and these also indicated that there was no difference in the sensitivity of phototrophs and heterotrophs. Therefore, the ecotoxicity threshold values were calculated using ecotoxicity data for both phototrophs and heterotrophs (Table S3) as recommended by Warne et al. (2018). The species sensitivity distribution and ecotoxicity threshold values for freshwater 2,4-D data (Figure S3 and Table S4, respectively) were calculated using Burrlioz V2 (CSIRO 2016).


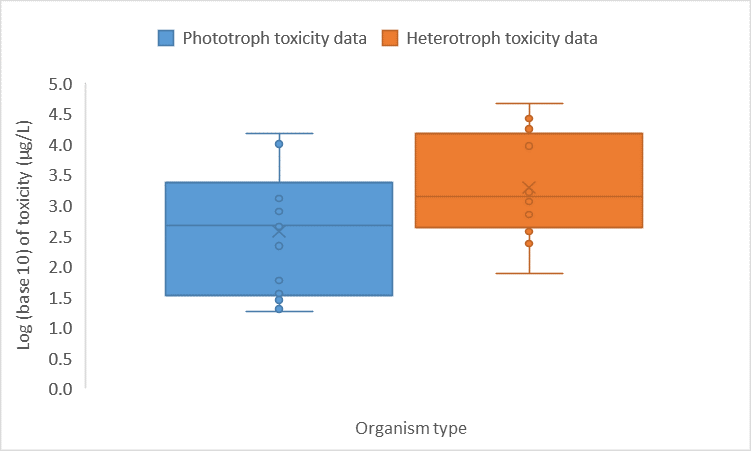


Figure S2. Box and whisker plot for 2,4-D freshwater toxicity to phototrophic and heterotrophic organisms.

Table S3. The chronic ecotoxicity data used to calculate the ecotoxicity threshold values for 2,4-D to freshwater species.

| **Species** | **Toxicity (µg/L)** |
| --- | --- |
| *Anabaena flos-aquae* | 1,266 |
| *Lemna gibba* | 215 |
| *Myriophyllum aquaticum* | 24.2 |
| *Navicula cryptotenella* | 500 |
| *Navicula pelliculosa* | 1,482 |
| *Selenastrum capricornutum* | 781 |
| *Daphnia magna* | 700 |
| *Pimephales promelas* | 1,799 |


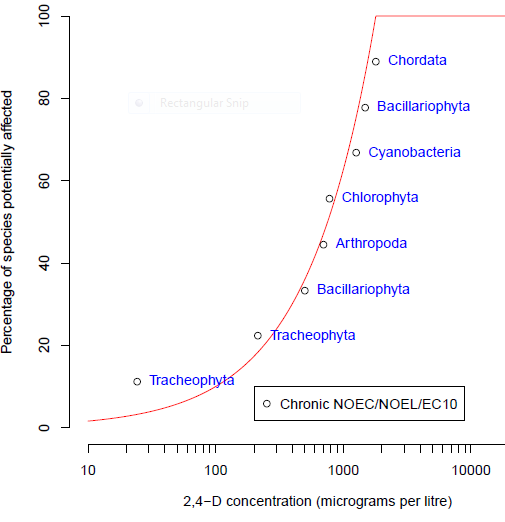


Figure S3. The species sensitivity distribution for chronic ecotoxicity data to 2,4-D.

Table S4. The ecotoxicity threshold values derived for 2,4-D to freshwater organisms.

| **Level of protection**  **(% species protected)** | **Concentration**  **(µg/L)** | **Concentration**  **(µmol/L)** |
| --- | --- | --- |
| 99 | 5.6 | 0.025 |
| 95 | 42 | 0.190 |
| 90 | 100 | 0.452 |
| 80 | 239 | 1.081 |

### Amicarbazone in freshwater

Amicarbazone is a triazolinone herbicide that exert effects by direct contact and via the soil. It inhibits the enzyme acetolactase synthase, which synthesises amino acids essential for plant metabolism and growth. As such it would be expected that amicarbazone would be more toxic to phototrophs than heterotrophs. The sensitivity of phototrophs and heterotrophs was compared following the weight of evidence approach recommended in Warne et al. (2018). The coefficient of bimodality test yielded a value of 0.436 indicating the distribution of sensitivities is unimodal (as the minimum value to indicate bimodality is 0.55). The phototrophs and heterotrophs data were also compared using a box and whiskers plot (Figure S4), which indicated that the groups were slightly separated. Given the distance of the bimodality coefficient value to the minimum cut-off value (i.e., 0.55) and the minimal separation of the toxicity data for the phototrophs and heterotrophs it was decided that the distribution was most likely unimodal. Therefore, toxicity data for all species (Table S5) were used to calculate the ETVs consistent with Warne et al. (2018). The SSD and ETVs for freshwater amicarbazone ecotoxicity data (Figure S5 and Table S6, respectively) were calculated using Burrlioz V2 (CSIRO 2016).


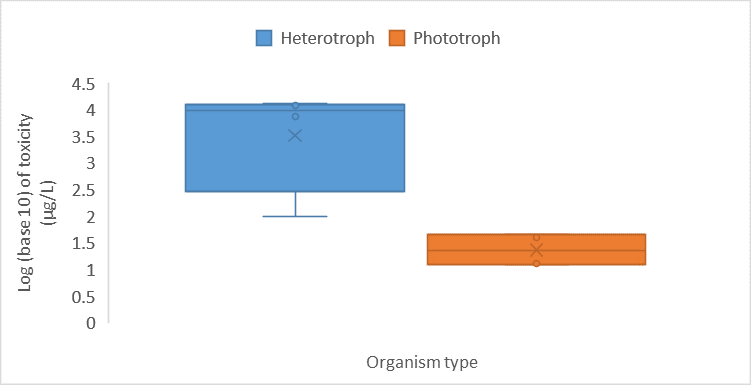


Figure S5. Box and whisker plot for amicarbazone freshwater toxicity to phototrophic and heterotrophic organisms.

Table S5. Toxicity data^1^ used to derive the ecotoxicity threshold value for amicarbazone to freshwater species.

| **Species** | **Toxicity (µg/L)** |
| --- | --- |
| *Anabaena flos-aquae* | 12.40 |
| *Daphnia magna* | 100.8 |
| *Lemna gibba* | 40.60 |
| *Navicula pelliculosa* | 46.20 |
| *Pimephales promelas* | 7,300 |
| *Pseudokirchneriella subcapitata* | 12.90 |

^1^ There were sufficient chronic NOEC and EC10 data for freshwater species to calculate ecotoxicity threshold values (ETVs) using the species sensitivity distribution method. However, the fit of the distribution to the data was poor so chronic NOEC, EC10 and converted chronic EC50 data were used.


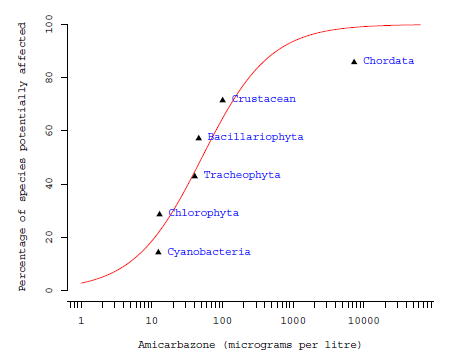


Figure S5. The species sensitivity distribution for chronic ecotoxicity data to amicarbazone.

Table S6. The ecotoxicity threshold values derived for amicarbazone to freshwater organisms.

| **Level of protection**  **(% species protected)** | **Concentration**  **(µg/L)** | **Concentration (µmol/L)** |
| --- | --- | --- |
| 99 | 0.32 | 0.00133 |
| 95 | 2 | 0.00829 |
| 90 | 4.5 | 0.0186 |
| 80 | 11 | 0.0456 |

### Asulam in freshwater

The available toxicity data for asulam from ECOTOX (US EPA, 2019b) and OPP (US EPA 2019c) are presented in Table S7. There were only sufficient toxicity data to derive a low reliability (interim) DGV using the Australian and New Zealand assessment factor method (Warne, 2001). Therefore, the lowest toxicity value of 3.12 mg/L (*Daphnia magna*) was divided by an assessment factor of 100 resulting in an ETV of 0.0312 mg/L or 31.2 µg/L. This equates to 0.136 µmol/L. All DGVs derived by the assessment factor method are now classed as having an unknown reliability (Warne et al., 2018).

Table S7. Toxicity data used to derive the ecotoxicity threshold value for asulam to freshwater species.

| **Species** | **Taxa** | **Purity / Grade** | **Duration**  **(hr)** | **Endpoint** | **Measure** | **Concentration (mg/L)** |
| --- | --- | --- | --- | --- | --- | --- |
| *Daphnia magna* | Crustacea | 88% | 96 | Immobile | EC50 | 67.00 |
| *Daphnia magna* | Crustacea | Tech | 96 | Immobile | EC50 | 3.12 |
| *Daphnia magna* | Crustacea | Tech | 48 | Immobile | EC50 | 27.06 |
| *Lepomis macrochirus* | Fish | 96.6% | 96 | Mortality | LC50 | >180 |
| *Lemna aequinoctialis* | Macrophyte | Analytical | 168 | Population growth rate | EC50 | 93.78 |
| *Oncorhynchus mykiss* | Fish | 88% | 96 | Mortality | LC50 | >175 |

### Bifenthrin in freshwater

Bifenthrin is a pyrethroid insecticide that affects the central and peripheral nervous system. Pyrethoids are highly toxic to insects and crustaceans (collectively called arthropods) but have a low toxicity to mammals. As such it would be expected that bifenthrin would be more toxic to arthropods than non-arthropods. The sensitivity of arthropods and non-arthropods was compared following the weight of evidence approach recommended in Warne et al. (2018). The coefficient of bimodality test yielded a value of 0.53 indicating the distribution of sensitivities is unimodal (as the minimum value to indicate bimodality is 0.55). The arthropods and non-arthropods data were also compared using a box and whiskers plot (Figure S6), which indicated that the groups were not separated. Given the bimodality coefficient value and the incomplete separation of the toxicity data for the arthropods and non-arthropods it was decided that the distribution was most likely unimodal. Therefore, chronic NOEC/EC10 toxicity data for all species (Table S8) were used to calculate the ETVs consistent with Warne et al. (2018). The SSD for freshwater bifenthrin ecotoxicity data (Figure S7) were calculated using Burrlioz V2 (CSIRO 2016).


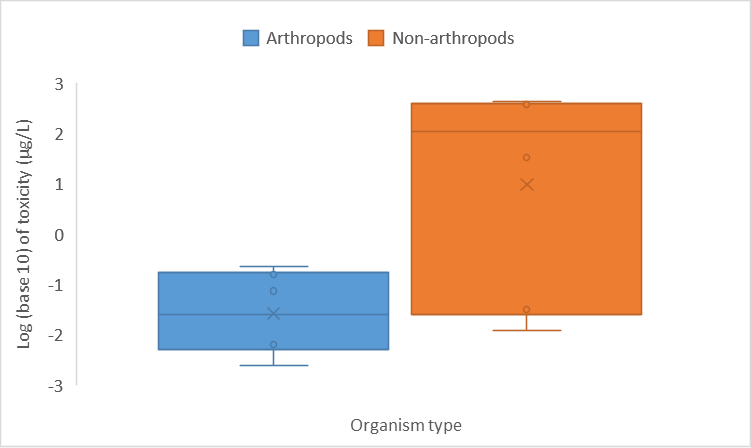


Figure S6. Box and whisker plot for bifenthrin freshwater toxicity to arthropod and non-arthropod organisms.

Table S8. Toxicity data (chronic NOEC/EC10) used to derive the ecotoxicity threshold value for bifenthrin to freshwater species.

| **Species** | **Toxicity (µg/L)** |
| --- | --- |
| *Anabaena flos-aquae* | 430 |
| *Ceriodaphnia dubia* | 0.227 |
| *Chironomus dilutus* | 0.00643 |
| *Hyalella azteca* | 0.00254 |
| *Navicula pelliculosa* | 370 |
| *Pseudokirchneriella subcapitata* | 380 |


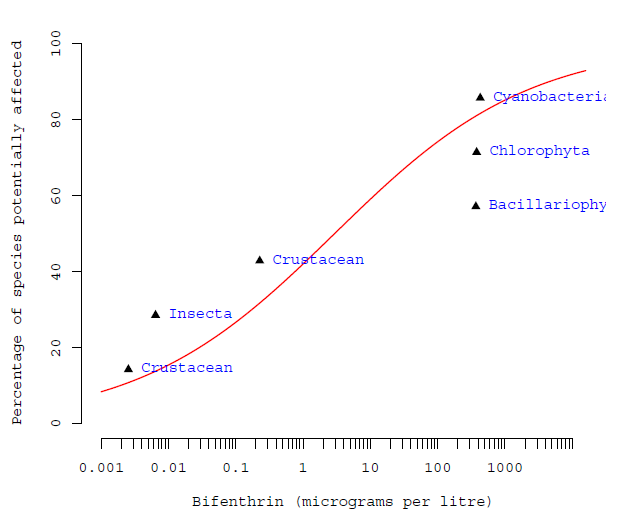


Figure S7. The species sensitivity distribution for chronic ecotoxicity data for arthropods and non-arthropods to bifenthrin.

The fit of the SSD to the chronic data for arthropods and non-arthropods is poor, particularly for the non-arthropod data. This combined with the mode of action and the proximity of the bimodality value to the significance cut-off suggested that the non-arthropod data should be removed. This was done, but in order to calculate the ETVs using the SSD method both chronic and converted acute data for arthropods were combined and used (Table S9). The resulting SSD and ETVs are presented in Figure S8 and Table S10, respectively.

Table S9. Toxicity data (chronic and converted acute) used to derive the ecotoxicity threshold value for bifenthrin to freshwater arthropod species.

| **Species** | **Toxicity (µg/L)** |
| --- | --- |
| *Ceriodaphnia dubia* | 0.227 |
| *Chironomus dilutus* | 0.00643 |
| *Chironomus tentans* | 0.0750 |
| *Daphnia magna* | 0.160 |
| *Hyalella azteca* | 0.00254 |
| *Procloeon sp.* | 0.00843 |


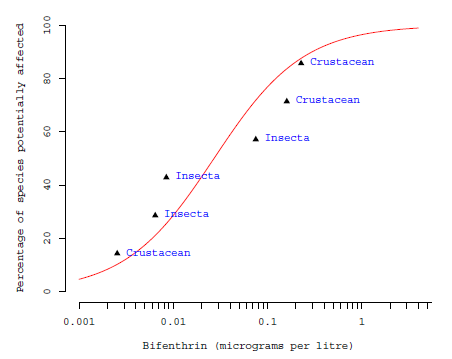


Figure S8. The species sensitivity distribution for chronic and converted acute ecotoxicity data for bifenthrin to freshwater arthropods.

Table S10. The ecotoxicity threshold values derived for bifenthrin to freshwater organisms.

| **Level of protection**  **(% species protected)** | **Concentration**  **(µg/L)** | **Concentration (µmol/L)** |
| --- | --- | --- |
| 99 | 0.00018 | 0.00000043 |
| 95 | 0.0011 | 0.0000026 |
| 90 | 0.0025 | 0.0000059 |
| 80 | 0.006 | 0.0000142 |

###

### Cadusafos in freshwater

Cadusafos is an organophosphate insecticide, specifically a nematicide. The available toxicity data for cadusafos from ECOTOX (US EPA 2019b) and OPP (US EPA 2019c) are presented in Table S11. There were only sufficient toxicity data to derive a low reliability (Environmental Concern Level) DGV using the Australian and New Zealand assessment factor method (Warne 2001). Therefore, the lowest acute toxicity value of 0.00182 mg/L (*Daphnia magna*) was divided by an assessment factor of 1000 resulting in an ETV of 0.00000182 mg/L or 0.00182 µg/L. This equates to 0.0000067 µmol/L. All DGVs derived by the assessment factor method are now classed as having an unknown reliability (Warne et al. 2018).

Table S11. Toxicity data used to derive the ecotoxicity threshold value for cadusafos to freshwater species.

| **Species** | **Taxa** | **Purity/ Grade** | **Duration** | **Endpoint** | **Measure** | **Concentration (mg/L)** |
| --- | --- | --- | --- | --- | --- | --- |
| *Daphnia magna* | Crustacea | 100% | 24 h | Immobile | EC50 | 0.00182 |
| *Lepomis macrochirus* | Fish | 100% | 4 d | Mortality | LC50 | 0.17 |
| *Oncorhynchus mykiss* | Fish | 100% | 4 d | Mortality | LC50 | 0.13 |

### Carbofuran in freshwater

Carbofuran is a carbamate insecticide that irreversibly inhibits acetylcholinesterase (AChE). As it is an insecticide it is expected to be more toxic to arthropods (which includes insects and crustaceans) than non-arthropods. The sensitivity of arthropods and non-arthropods were compared following the weight of evidence approach recommended in Warne et al. (2018). The coefficient of bimodality test yielded a value of 0.27 indicating the distribution of sensitivities is unimodal (as the minimum value to indicate bimodality is 0.55). The arthropods and non-arthropods data were also compared using a box and whiskers plot (Figure S9), which indicated that the groups were not separated. Given the distance of the bimodality coefficient value to the minimum cut-off value (i.e., 0.55) and the incomplete separation of the toxicity data for the arthropods and non-arthropods it was decided that the distribution was most likely unimodal. Therefore, toxicity data for all species (Table S12) were used to calculate the ETVs consistent with Warne et al. (2018). Both chronic and converted acute data were used as there were insufficient chronic data alone to use the SSD method. The SSD and ETVs for freshwater carbofuran ecotoxicity data (Figure S10 and Table S13, respectively) were calculated using Burrlioz V2 (CSIRO 2016).


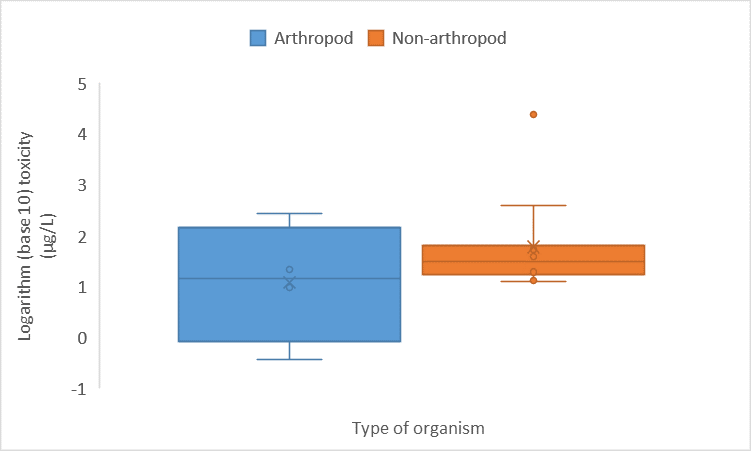


Figure S9. Box and whisker plot for carbofuran freshwater toxicity to arthropod and non-arthropod organisms.

Table S12. The ecotoxicity data used to calculate the ecotoxicity threshold values for carbofuran to freshwater species.

| **Species** | **Toxicity (µg/L)** |
| --- | --- |
| *Daphnia magna* | 9.80 |
| *Gammarus pseudolimnaeus* | 0.38 |
| *Ictalurus punctatus* | 24.8 |
| *Lemna minor* | 23,600 |
| *Lepomis macrochirus* | 12.57 |
| *Morone saxatilis* | 19.32 |
| *Oncorhynchus kisutch* | 53.00 |
| *Oncorhynchus mykiss* | 24.80 |
| *Ophiogomphus sp.* | 22.00 |
| *Oreochromis niloticus* | 39.25 |
| *Perca flavescens* | 13.28 |
| *Pimephales promelas* | 67.60 |
| *Procambarus clarkii* | 270 |
| *Pseudokirchneriella subcapitata* | 400 |
| *Salmo trutta* | 56.00 |
| *Salvelinus namaycush* | 16.40 |


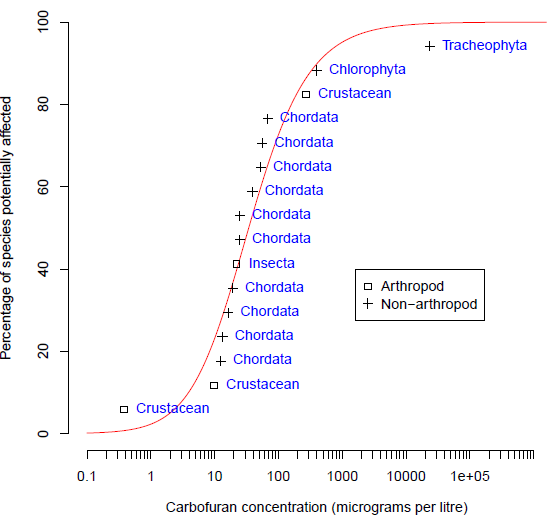


Figure S10. The species sensitivity distribution for chronic ecotoxicity data for carbofuran to all available freshwater species.

Table S13. The ecotoxicity threshold values derived for carbofuran to freshwater organisms.

| **Level of protection**  **(% species protected)** | **Concentration**  **(µg/L)** | **Concentration (µmol/L)** |
| --- | --- | --- |
| 99 | 0.49 | 0.0022 |
| 95 | 2.0 | 0.0090 |
| 90 | 3.9 | 0.018 |
| 80 | 8.3 | 0.038 |

### Chlorpyrifos in freshwater

Chlorpyrifos is an organophosphate insecticide that inhibits the activity of the enzyme acetylcholinesterase, leading to hyper-stimulation of muscles, the depletion of energy and rapidly to death. As an insecticide it is expected that chlorpyrifos will be more toxic to insects and other arthropods (e.g. crustaceans) than to non-arthropods. Water quality guidelines were derived for chlorpyrifos in 2000 (ANZECC and ARMCANZ 2000) and were not included in the revision of the guidelines. Therefore, data from 2000 to 2020 were collected from the ECOTOX (US EPA 2019b) and OPP (US EPA 2019c) databases to derive ETVs for chlorpyrifos. The sensitivity of arthropods and non-arthropods were compared following the weight of evidence approach recommended in Warne et al. (2018). The coefficient of bimodality test yielded a value of 0.45 indicating the distribution of sensitivities is unimodal (as the minimum value to indicate bimodality is 0.55). The arthropods and non-arthropods data were also compared using a box and whiskers plot (Figure S11) and a species sensitivity distribution with chronic and converted acute data (Figure S12). This indicated that the groups were not separated. Given the distance of the bimodality coefficient value to the minimum cut-off value (i.e., 0.55) and the incomplete separation of the toxicity data for the arthropods and non-arthropods it was decided that the distribution was most likely unimodal. Therefore, chronic toxicity data for all species (Table S14) were used to calculate the ETVs consistent with Warne et al. (2018). The species sensitivity distribution and ETVs for freshwater chlorpyrifos data (Figure S13 and Table S15, respectively) were calculated using Burrlioz V2 (CSIRO 2016).


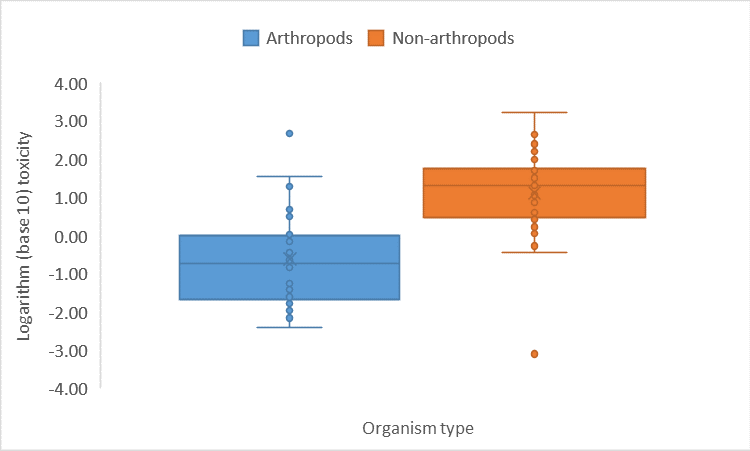


Figure S11. Box and whisker plots of chlorpyrifos toxicity to freshwater arthropods and non-arthropods.


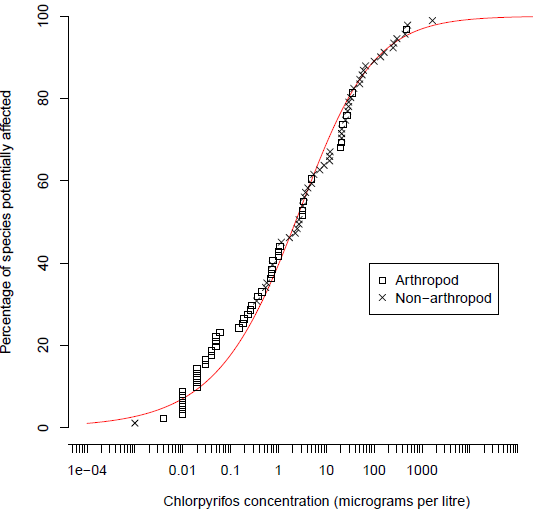


Figure S12. The species sensitivity distribution for all ecotoxicity data for chlorpyrifos to all available freshwater species that was used to assess the modality of the data.

Table S14. The ecotoxicity data used to calculate the ecotoxicity threshold values for chlorpyrifos to freshwater species.

| **Species** | **Toxicity (µg/L)** |
| --- | --- |
| *Anaxyrus americanus* | 21 |
| *Asellus aquaticus* | 21 |
| *Ceriodaphnia dubia* | 0.02 |
| *Chironomus riparius* | 1 |
| *Chironomus tentans* | 0.02 |
| *Chlorella sp.* | 258 |
| *Crangonyx pseudogracilis* | 1.08 |
| *Daphnia carinata* | 0.025 |
| *Daphnia magna* | 0.18 |
| *Daphnia pulex* | 0.014 |
| *Hyla versicolor* | 2.7 |
| *Lampsilis siliquoidea* | 12 |
| *Leptodiaptomus minutus* | 3.2 |
| *Lithobates clamitans* | 21 |
| *Lithobates pipiens* | 3.2 |
| *Lithobates sylvaticus* | 21 |
| *Melanotaenia fluviatilis* | 9 |
| *Menidia beryllina* | 0.75 |
| *Nitzschia sp.* | 443 |
| *Oncorhynchus tshawytscha* | 3.7 |
| *Pimephales promelas* | 0.57 |
| *Poecilia reticulata* | 0.0008 |
| *Pseudacris crucifer* | 2.7 |
| *Pseudacris regilla* | 50 |
| *Pseudacris sierra* | 100 |
| *Rana boylii* | 50 |
| *Rana dalmatina* | 56.1 |
| *Rana sierrae* | 500 |
| *Skistodiaptomus oregonensis* | 3.2 |
| *Tandanus tandanus* | 11.7 |


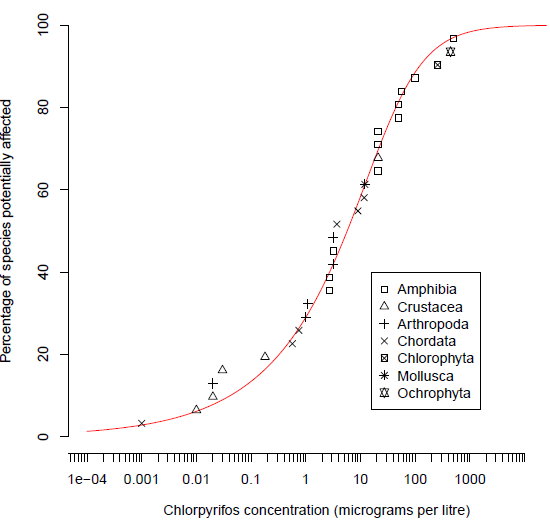


Figure S13. The species sensitivity distribution for chronic NOEC/EC10 and chronic LOEC/EC50 ecotoxicity data for chlorpyrifos to all chronic freshwater species.

Table S15. The ecotoxicity threshold values derived for chlorpyrifos to freshwater organisms.

| **Level of protection**  **(% species protected)** | **Concentration**  **(µg/L)** | **Concentration (µmol/L)** |
| --- | --- | --- |
| 99 | 0.000047 | 0.00000013 |
| 95 | 0.0054 | 0.0000154 |
| 90 | 0.042 | 0.000120 |
| 80 | 0.33 | 0.000941 |

### Dicamba in freshwater

Dicamba is a synthetic auxin herbicide. Synthetic auxins mimic the plant hormone, auxin (indolylacetic acid, or IAA), which promotes stem elongation. Synthetic auxins cause cells to elongate in an uncontrolled and disorganised manner, leading to plant death. As such dicamba would be expected to more toxic to plants (particularly dicots) than animals. The sensitivity of phototrophs and heterotrophs was compared following the weight of evidence approach recommended in Warne et al. (2018). The coefficient of bimodality test yielded a value of 0.34 indicating the distribution of sensitivities is unimodal (as the minimum value to indicate bimodality is 0.55). The phototrophs and heterotrophs data were also compared using a box and whiskers plot (Figure S14). These analyses indicated that there was an incomplete separation between the two groups. Given the distance of the bimodality coefficient value to the minimum cut-off value (i.e., 0.55) and the incomplete separation of the toxicity data for the phototrophs and heterotrophs it was decided that the distribution was most likely unimodal. Therefore, toxicity data for all species (Table S16) were used to calculate the ecotoxicity thresholds consistent with Warne et al. (2018). The species sensitivity distribution and ecotoxicity threshold values for freshwater dicamba data (Figure S15 and Table S17, respectively) were calculated using Burrlioz V2 (CSIRO 2016).


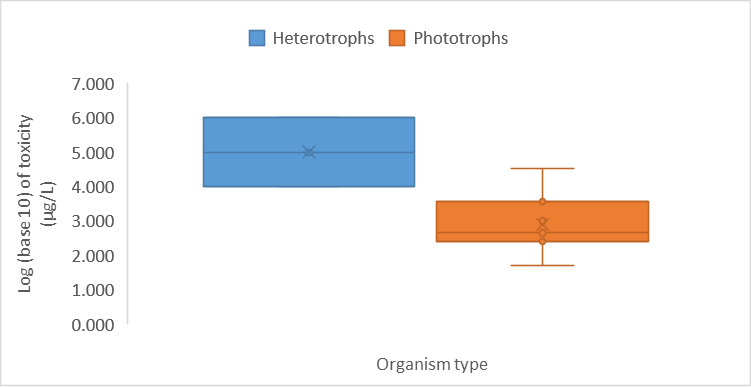


Figure S14. Box and whisker plots for dicamba freshwater toxicity to phototrophic and heterotrophic organisms.

Table S16. The ecotoxicity data used to calculate the ecotoxicity threshold values for dicamba to freshwater species.

| **Species** | **Toxicity (µg/L)** |
| --- | --- |
| *Anabaena flos-aquae* | 32,000 |
| *Cyprinus carpio* | 10,000 |
| *Daphnia magna* | 97,000 |
| *Lemna gibba* | 250 |
| *Lemna minor* | 1,000 |
| *Myriophyllum aquaticum* | 52 |
| *Myriophyllum spicatum* | 450 |
| *Navicula pelliculosa* | 260 |
| *Oncorhynchus mykiss* | 1,000,000 |
| *Pseudokirchneriella subcapitata* | 3,700 |


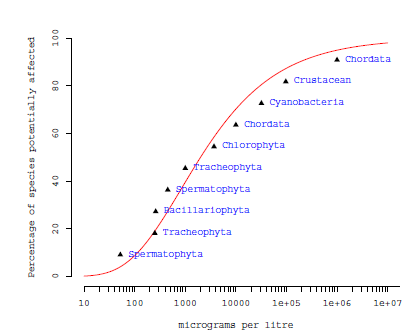


Figure S15. The species sensitivity distribution for chronic ecotoxicity data for dicamba to all available freshwater species.

Table S17. The ecotoxicity threshold values derived for dicamba to freshwater organisms.

| **Level of protection**  **(% species protected)** | **Concentration**  **(µg/L)** | **Concentration (µmol/L)** |
| --- | --- | --- |
| 99 | 22 | 0.100 |
| 95 | 61 | 0.276 |
| 90 | 114 | 0.516 |
| 80 | 268 | 1.212 |

### Diquat dibromide in freshwater

The available chronic toxicity data for diquat dibromide from ECOTOX (US EPA, 2019b) and OPP (US EPA 2019c) are presented in Table S18. There were only sufficient toxicity data to derive a low reliability (Environmental Concern Level) DGV using the Australian and New Zealand assessment factor method (Warne, 2001). Therefore, the lowest toxicity value (which was a chronic value) of 0.005 mg/L (*Pseudokirchneriella subcapitata*) was divided by an assessment factor of 200 resulting in an ETV of 0.000025 mg/L or 0.025 µg/L. This equates to 0.0000712 µmol/L. All DGVs derived by the assessment factor method are now classed as having an unknown reliability (Warne et al., 2018).

Table S18. Chronic toxicity data used to derive the ecotoxicity threshold value for diquat dibromide to freshwater species.

| **Species** | **Taxa** | **Purity/**  **Grade** | **Duration** | **Endpoint** | **Measure** | **Concentration (mg/L)** |
| --- | --- | --- | --- | --- | --- | --- |
| *Myriophyllum sibiricum* | Macrophyte | 99% | 14 d | Growth (area, whole organism) | NOEC | 0.0117 |
| *Pseudokirchneriella subcapitata* | Green algae | Tech | 96 h | Abundance | EC50 | 0.034 |
| *Pseudokirchneriella subcapitata* | Green algae | Tech | 96 h | Abundance | EC50 | 0.005 |

### Fipronil in freshwater

Based on the current understanding of the mode of action of fipronil, it would be expected that arthropods (insects and crustaceans) would be more sensitive than other organisms as it is a GABA- and glutamate-gated chloride channel antagonist, and glutamate receptors are insect specific. The sensitivity of arthropods and non-arthropods was compared following the weight of evidence approach recommended in Warne et al. (2018). The coefficient of bimodality test yielded a value of 0.51 indicating the distribution of sensitivities is unimodal (as the minimum value to indicate bimodality is 0.55). The arthropod and non-arthropod data were also compared using a box and whiskers plot (Figure S16) and a species sensitivity distribution (Figure S17). These indicated that there was a marked but incomplete separation between the two groups. Given the specific mode of action and the marked but incomplete separation in sensitivity it was decided that the distribution was most likely bimodal. Therefore, only chronic arthropod data (Table S19) were used to calculate the ecotoxicity thresholds consistent with Warne et al. (2018).


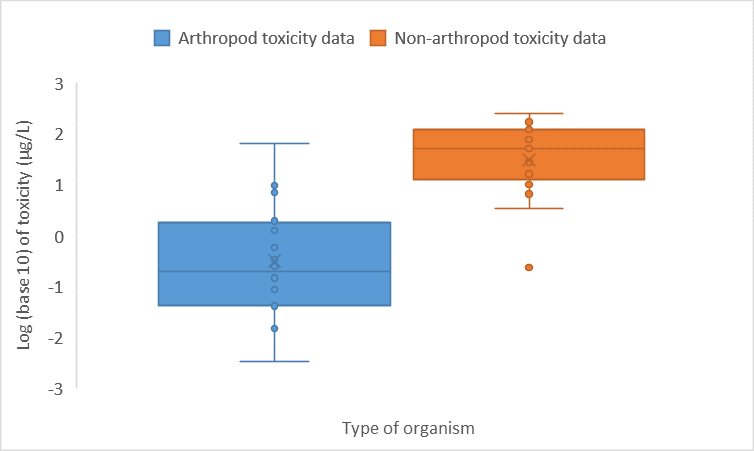


Figure S16. Box and whisker plots for fipronil freshwater and marine toxicity to arthropod and non-arthropod organisms.


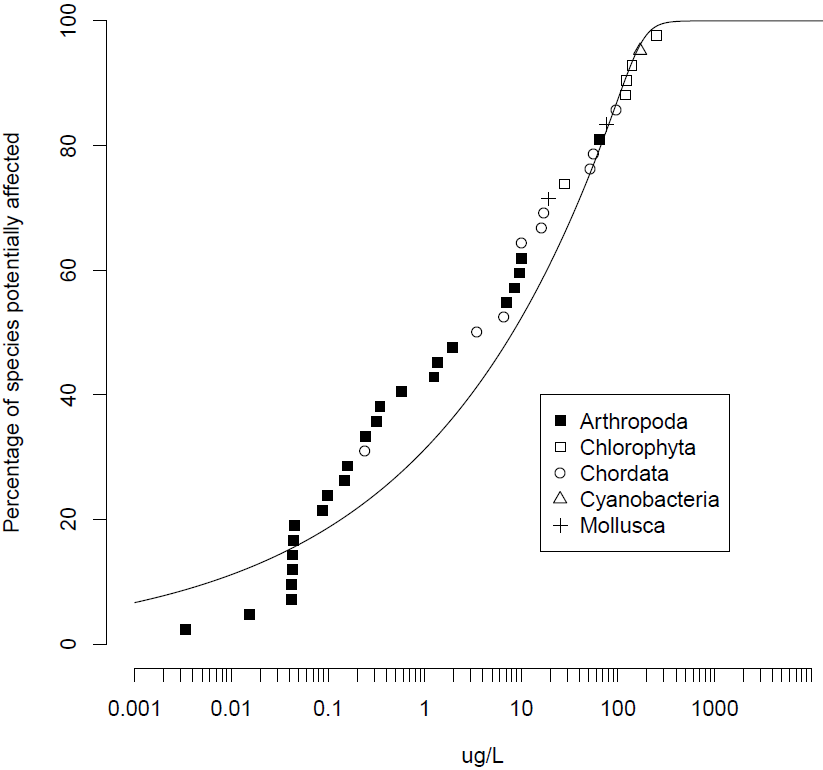


Figure S17. Species sensitivity distribution, generated by Burrlioz 2.0, for fipronil freshwater and marine toxicity to arthropod and non-arthropod organisms.

The species sensitivity distribution and ecotoxicity threshold values for freshwater fipronil data were calculated using Burrlioz V2 (CSIRO 2016) and are presented in Figure S18 and Table S20, respectively.

Table S19. The ecotoxicity data for arthropods used to calculate the ecotoxicity threshold values for fipronil to freshwater species.

| **Species** | **Toxicity (µg/L)** |
| --- | --- |
| *Ceriodaphnia dubia* | 10.00 |
| *Daphnia magna* | 9.60 |
| *Acanthocyclops robustus* | 8.489 |
| *Aedes aegypti* | 0.319 |
| *Aedes albopictus HAmAal strain* | 1.365 |
| *Aedes taeniorhynchus* | 0.043 |
| *Anopheies quadrimaculatus* | 0.043 |
| *Chaoborus crystallinus* | 64.633 |
| *Cheumatopsyche brevilineata* | 0.015 |
| *Chironomus annularius* | 0.245 |
| *Chironomus crassicaudatus* | 0.042 |
| *Culex nigripalpus* | 0.087 |
| *Culex quinqefasciatus* | 0.579 |
| *Diaptomus castor* | 0.345 |
| *Glyptotendipes paripes* | 0.042 |
| *Hexagenia sp.* | 0.044 |
| *Polypedilum nubiferum* | 0.148 |
| *Procambarus clarkii* | 6.975 |
| *Procambarus zonangulus* | 1.95 |
| *Simocephalus elizabethae* | 1.253 |


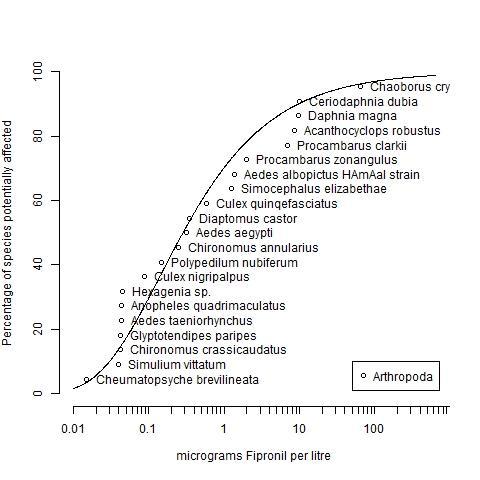


Figure S18. The species sensitivity distribution for chronic arthropod ecotoxicity data to fipronil.

Table S20. The ecotoxicity threshold values derived for fipronil to freshwater organisms.

| **Level of protection (% species protected)** | **Concentration (µg/L)** | **Concentration (µmol/L)** |
| --- | --- | --- |
| 99 | 0.0089 | 0.0000204 |
| 95 | 0.018 | 0.000041 |
| 90 | 0.032 | 0.000073 |
| 80 | 0.061 | 0.00014 |

### Fluazifop-P-butyl in freshwater

The available toxicity data for fluazifop-P-butyl from ECOTOX (US EPA 2019b) and OPP (US EPA 2019c) are presented in Table S21. There were only sufficient toxicity data to derive a low reliability (Environmental Concern Level) DGV using the Australian and New Zealand assessment factor method (Warne 2001). Therefore, the lowest toxicity value (which was a chronic value) of 0.88 mg/L (*Pseudokirchneriella subcapitata*) was divided by an assessment factor of 200 resulting in an ETV of 0.0044 mg/L or 4.4 µg/L. This equates to 0.0115 µmol/L. All DGVs derived by the assessment factor method are now classed as having an unknown reliability (Warne et al. 2018).

Table S21. Toxicity data used to derive the ecotoxicity threshold value for fluazifop-P-butyl to freshwater species.

| **Species** | **Taxa** | **Purity/Grade** | **Duration** | **Endpoint** | **Measure** | **Concentration (mg/L)** |
| --- | --- | --- | --- | --- | --- | --- |
| *Anabaena flos-aquae* | Blue-Green algae | 95.4% | 96 hr | N/A | NOEL | 48 |
| *Lemna gibba* | Macrophyte | 94% | 14 d | N/A | NOEL | 1.4 |
| *Pseudokirchneriella subcapitata* | Green algae | 81.3% | 4 d | Abundance | NOEL | 0.88 |

### Fluensulfone in freshwater

Fluensulfone is a nematicide that inhibits development, egg-laying and hatching, feeding and movement of nematodes. It would be expected that fluensulfone would be more toxic to nematodes than to other organisms. The sensitivity of fluensulfone to all available species was compared following the weight of evidence approach recommended in Warne et al. (2018). The coefficient of bimodality test yielded a value of 0.321 indicating the distribution of sensitivities is unimodal (as the minimum value to indicate bimodality is 0.55). This was supported by the SSD of all species with chronic and converted acute data (Figure S19) which shows no marked difference in the sensitivities of different organism types. However, there were no nematode toxicity data in the ECOTOX database or the OPP database that passed the data screening and quality checking. Given this, it was decided to use all chronic NOEC/EC10 data irrespective of the type of organism to calculate the ecotoxicity thresholds consistent with Warne et al. (2018).


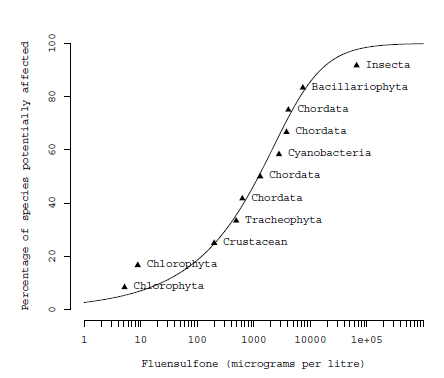


Figure S19. Species sensitivity distribution, generated by Burrlioz 2.0, for fluensulfone freshwater toxicity to all organisms.

The chronic toxicity data that was used to calculate ETVs for fluensulfone to freshwater organisms are presented in Table S22. The species sensitivity distribution and ecotoxicity threshold values for chronic NOEC/EC10 toxicity data for all freshwater organisms were calculated using Burrlioz V2 (CSIRO 2016) and are presented in Figure S20 and Table S23, respectively.

Table S22. The chronic NOEC/EC10 ecotoxicity data used to calculate the ecotoxicity threshold values for fluensulfone to freshwater species.

| **Species** | **Toxicity (µg/L)** |
| --- | --- |
| *Anabaena flos-aquae* | 2,800 |
| *Chironomus dilutus* | 66,000 |
| *Daphnia magna* | 200 |
| *Lemna gibba* | 490 |
| *Navicula pelliculosa* | 7,400 |
| *Pimephales promelas* | 630 |
| *Pseudokirchneriella subcapitata* | 8.9 |


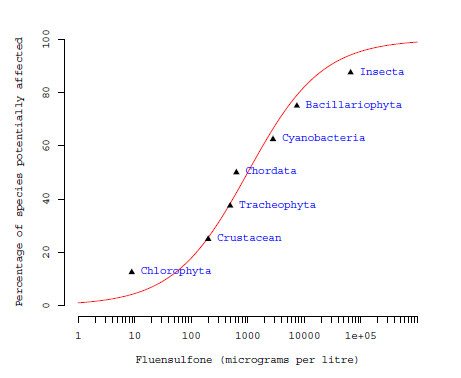


Figure S20. The species sensitivity distribution for chronic freshwater ecotoxicity data to fluensulfone.

Table S23. The ecotoxicity threshold values derived for fluensulfone to freshwater organisms.

| **Level of protection (% species protected)** | **Concentration (µg/L)** | **Concentration (µmol/L)** |
| --- | --- | --- |
| 99 | 0.98 | 0.00336 |
| 95 | 12 | 0.0411 |
| 90 | 37 | 0.127 |
| 80 | 124 | 0.425 |

### Flumioxazin in freshwater

Flumioxazin is a light-dependent peroxidising herbicide (LDPH). It exerts its toxicity by blocking the synthesis of heme and chlorophyll which results in the accumulation of photo-toxic porphyrins. As such it is expected that flumioxazin would be more toxic to phototrophs than to heterotrophs. The sensitivity of phototrophs and heterotrophs was compared following the weight of evidence approach recommended in Warne et al. (2018). The coefficient of bimodality test yielded a value of 0.325 indicating the distribution of sensitivities is unimodal (as the minimum value to indicate bimodality is 0.55). The phototrophs and heterotrophs data were also compared using a box and whiskers plot (Figure S21) and a species sensitivity distribution (Figure S22). These indicated that there was an incomplete separation between the two groups. Given the specific mode of action and the marked but incomplete separation in sensitivity, it was decided that the distribution was most likely unimodal. Therefore, chronic NOEC/EC10 data for all species (Table S24) were used to calculate the ecotoxicity thresholds consistent with Warne et al. (2018).


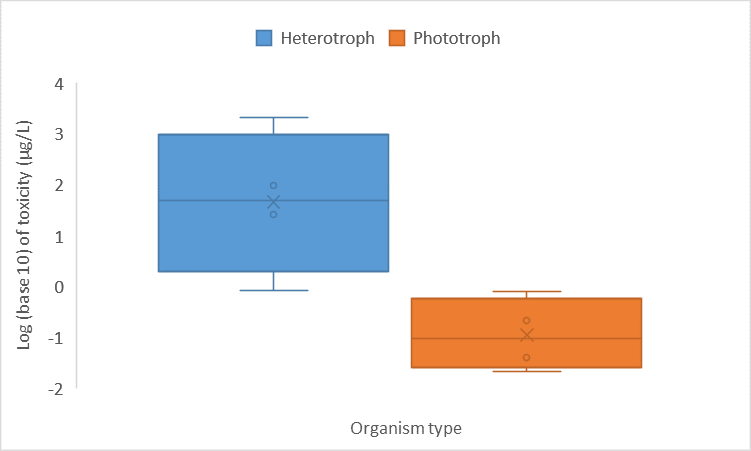


Figure S21. Box and whisker plot for flumioxazin toxicity to freshwater phototrophic and heterotrophic organisms.


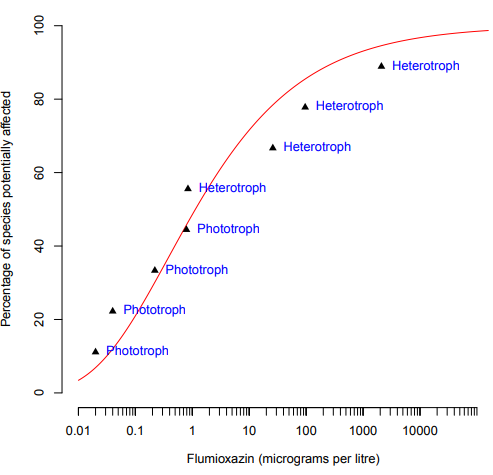


Figure S22. The species sensitivity distribution for chronic ecotoxicity data for flumioxazin to all available freshwater species.

Table S24. The chronic NOEC/EC10 ecotoxicity data used to calculate the ecotoxicity threshold values for flumioxazin to all freshwater species.

| **Species** | **Toxicity (µg/L)** |
| --- | --- |
| *Anabaena flos-aquae* | 0.02 |
| *Daphnia magna* | 26.00 |
| *Lemna gibba* | 0.22 |
| *Navicula pelliculosa* | 0.04 |
| *Oncorhynchus mykiss* | 96.12 |
| *Pimephales promelas* | 0.84 |
| *Pseudokirchneriella subcapitata* | 0.79 |

The species sensitivity distribution and ecotoxicity threshold values for chronic NOEC/EC10 toxicity data for all freshwater organisms were calculated using Burrlioz V2 (CSIRO 2016) and are presented in Figure S23 and Table S25, respectively.


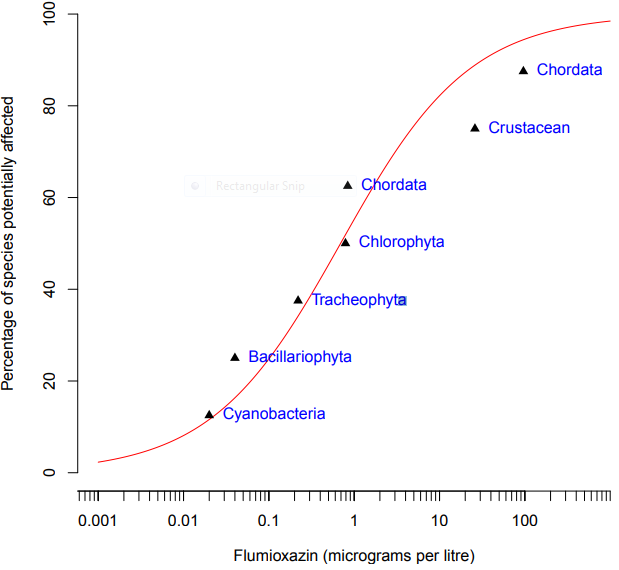


Figure S23. The species sensitivity distribution for chronic ecotoxicity data for flumioxazin to all available freshwater species that were used to derive the ecotoxicity threshold values.

Table S25. The ecotoxicity threshold values derived for flumioxazin to freshwater organisms.

| **Level of protection (% species protected)** | **Concentration (µg/L)** | **Concentration (µmol/L)** |
| --- | --- | --- |
| 99 | 0.00023 | 0.000001 |
| 95 | 0.0041 | 0.000012 |
| 90 | 0.015 | 0.000042 |
| 80 | 0.062 | 0.000175 |

### Flutriafol in freshwater

The available chronic toxicity data for flutriafol from ECOTOX (US EPA, 2019b) and OPP (US EPA 2019c) are presented in Table S26. There were only sufficient toxicity data to derive a low reliability (Interim) DGV (Warne, 2001). Therefore, the lowest toxicity value (which was a chronic value) of 0.31 mg/L (*Daphnia magna*) was divided by an assessment factor of 20 resulting in an ETV of 0.0155 mg/L or 15.5 µg/L. This equates to 0.0514 µmol/L. All DGVs derived by the assessment factor method are now classed as having an unknown reliability (Warne et al., 2018).

Table S26. Chronic toxicity data used to derive the ecotoxicity threshold value for flutriafol to freshwater species.

| **Species** | **Taxa** | **Purity/Grade** | **Duration** | **Endpoint** | **Measure** | **Concentration (mg/L)** |
| --- | --- | --- | --- | --- | --- | --- |
| *Daphnia magna* | Crustacea | 95.5% | 21 d | Growth | NOEL | 0.31 |
| *Daphnia magna* | Crustacea | 94.4% | 21 d | Reproduction | NOEL | 1 |
| *Lemna gibba* | Macrophyte | 95.1% | 7 d | N/A | NOEL | 0.57 |
| *Pimephales promelas* | Fish | 95.1% | 33 d | Survival | NOEL | 4.8 |

### Glufosinate ammonium in freshwater

Glufosinate ammonium is a herbicide that irreversibly inhibits glutamine synthetase, so it is expected to be more toxic to phototrophs than heterotrophs. The sensitivity of phototrophs and heterotrophs was compared following the weight of evidence approach recommended in Warne et al. (2018). The coefficient of bimodality test yielded a value of 0.43 indicating the distribution of sensitivities is unimodal (as the minimum value to indicate bimodality is 0.55). The phototrophs and heterotrophs data were also compared using a box and whiskers plot (Figure S24), which indicated that the groups were not separated. Given the distance of the bimodality coefficient value to the minimum cut-off value (i.e., 0.55) and the incomplete separation of the toxicity data for the phototrophs and heterotrophs it was decided that the distribution was most likely unimodal. Therefore, chronic toxicity data for all species (Table S27) were used to calculate the ETVs consistent with Warne et al. (2018). The SSD and ETVs for freshwater glufosinate ammonium toxicity data (Figure S25 and Table S28, respectively) were calculated using Burrlioz V2 (CSIRO 2016).


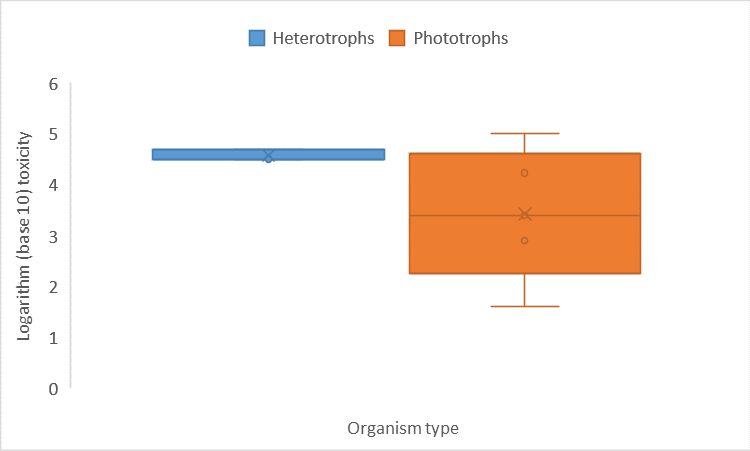


Figure S24. Box and whisker plots for glufosinate ammonium chronic freshwater toxicity to phototrophic and heterotrophic organisms.

Table S27. The chronic ecotoxicity data used to calculate the ecotoxicity threshold values for glufosinate ammonium to freshwater species.

| **Species** | **Toxicity (µg/L)** |
| --- | --- |
| *Anabaena flos-aquae* | 41 |
| *Daphnia magna* | 32,000 |
| *Lemna gibba* | 800 |
| *Navicula pelliculosa* | 17,000 |
| *Oncorhynchus mykiss* | 50,000 |
| *Pseudokirchneriella subcapitata* | 2,500 |
| *Scenedesmus subspicatus* | 100,000 |


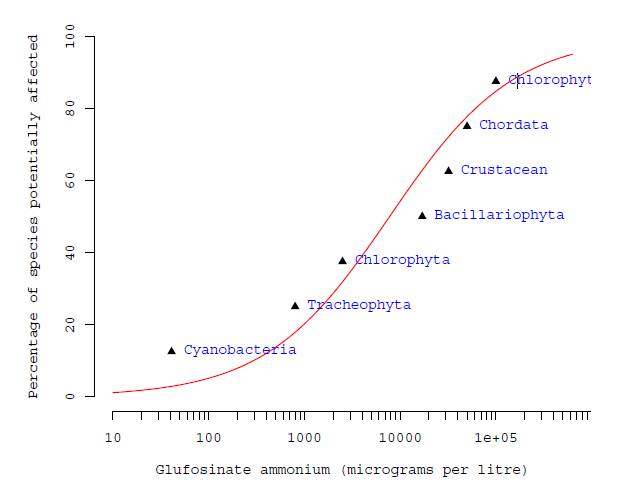


Figure S25. The species sensitivity distribution for chronic ecotoxicity data for glufosinate ammonium to all available freshwater species.

Table S28. The ecotoxicity threshold values derived for glufosinate ammonium to freshwater organisms.

| **Level of protection**  **(% species protected)** | **Concentration**  **(µg/L)** | **Concentration (µmol/L)** |
| --- | --- | --- |
| 99 | 8.2 | 0.041 |
| 95 | 96 | 0.484 |
| 90 | 293 | 1.479 |
| 80 | 982 | 4.956 |

### Halosulfuron – methyl in freshwater

Halosulfuron – methyl is a sulfonylurea herbicide that inhibits the enzyme acetolactate synthase that synthesises amino acids, so it is expected to be more toxic to phototrophs than heterotrophs. The sensitivity of phototrophs and heterotrophs were compared following the weight of evidence approach recommended in Warne et al. (2018). The coefficient of bimodality test yielded a value of 0.29 indicating the distribution of sensitivities is unimodal (as the minimum value to indicate bimodality is 0.55). The phototrophs and heterotrophs data were also compared using a box and whiskers plot (Figure S26), which indicated that the groups were not separated. Given the distance of the bimodality coefficient value to the minimum cut-off value (i.e., 0.55) and the incomplete separation of the toxicity data for the phototrophs and heterotrophs it was decided that the distribution was most likely unimodal. Therefore, chronic toxicity data for all species (Table S29) were used to calculate the ETVs consistent with Warne et al. (2018). The SSD and ETVs for freshwater halosulfuron – methyl toxicity data (Figure S27 and Table S30, respectively) were calculated using Burrlioz V2 (CSIRO 2016).


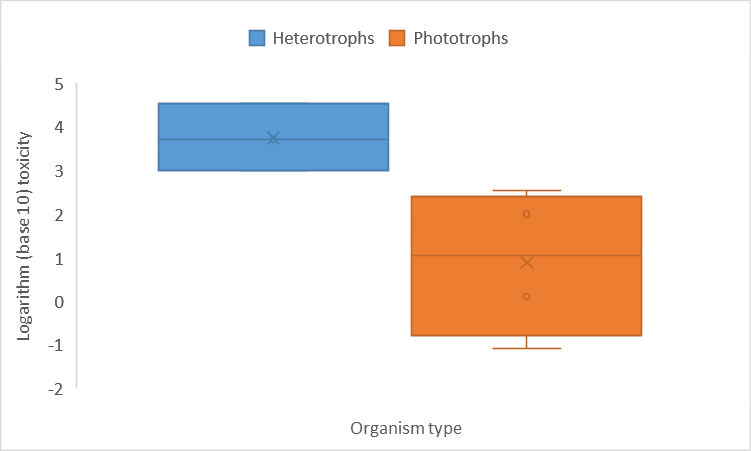


Figure S26. Box and whisker plot for halosulfuron – methyl chronic freshwater toxicity to phototrophic and heterotrophic organisms.

Table S29. The chronic ecotoxicity data used to calculate the ecotoxicity threshold values for halosulfuron – methyl to freshwater species.

| **Species** | **Toxicity (µg/L)** |
| --- | --- |
| *Anabaena flos-aquae* | 100 |
| *Daphnia magna* | 980 |
| *Lemna gibba* | 0.08 |
| *Navicula pelliculosa* | 350 |
| *Oncorhynchus mykiss* | 34,000 |
| *Pseudokirchneriella subcapitata* | 1.3 |


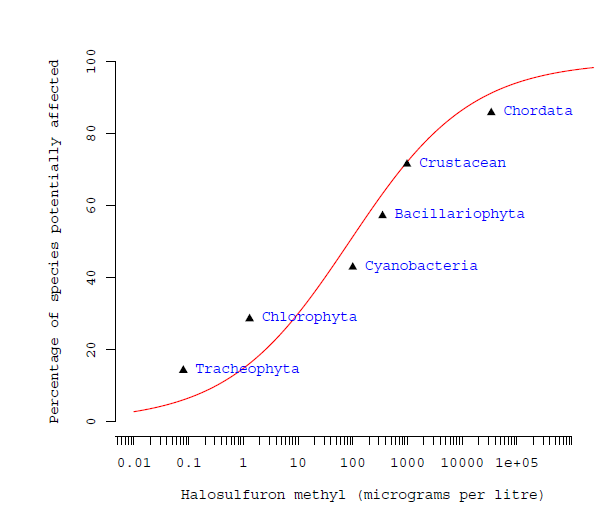


Figure S27. The species sensitivity distribution for chronic ecotoxicity data for halosulfuron – methyl to all available freshwater species.

Table S30. The ecotoxicity threshold values derived for halosulfuron – methyl to freshwater organisms.

| **Level of protection**  **(% species protected)** | **Concentration**  **(µg/L)** | **Concentration (µmol/L)** |
| --- | --- | --- |
| 99 | 0.00069 | 0.00000159 |
| 95 | 0.047 | 0.000108 |
| 90 | 0.32 | 0.000736 |
| 80 | 2.5 | 0.00575 |

### MSMA in freshwater

The available toxicity data for MSMA from ECOTOX (US EPA 2019b) and OPP (US EPA 2019c) are presented in Table S31. There was only one toxicity value for freshwater organisms. This permitted the derivation of a low reliability (Environmental Concern Level) DGV (Warne 2001). Therefore, the toxicity value (which was a chronic value) of 0.163 mg/L (Chlorella pyrenoidosa) was divided by an assessment factor of 1000 resulting in an ETV of 0.000163 mg/L or 0.163 µg/L. This equates to 0.001 µmol/L. All DGVs derived by the assessment factor method are now classed as having an unknown reliability (Warne et al. 2018).

Table S31. Toxicity data used to calculate the ecotoxicity threshold value for MSMA to freshwater species.

| **Species** | **Taxa** | **Purity/Grade** | **Duration** | **Endpoint** | **Measure** | **Concentration (mg/L)** |
| --- | --- | --- | --- | --- | --- | --- |
| *Chlorella pyrenoidosa* | Green algae | >98% | 21 d | Population growth rate | NOEC | 0.16296 |

### S-metolachlor in freshwater

Metolachlor is a herbicide that inhibits protein synthesis and s-metolachlor is the active enantiomer (form) of metolachlor. Metolachlor used to be a mixture of the R and S enantiomer forms of metolachlor but is now predominantly the S-metolachlor enantiomer. The sensitivity of phototrophs and heterotrophs was compared following the weight of evidence approach recommended in Warne et al. (2018). The coefficient of bimodality test yielded a value of 0.46 indicating the distribution of sensitivities is unimodal (as the minimum value to indicate bimodality is 0.55). The phototrophs and heterotrophs data were also compared using a box and whiskers plot (Figure S28) and a species sensitivity distribution with both chronic and converted acute data (Figure S29). These indicated that there was an incomplete separation between the two groups. Given the distance of the bimodality coefficient value to the minimum cut-off value (i.e., 0.55) and the incomplete separation of the toxicity data for the phototrophs and heterotrophs it was decided that the distribution was most likely unimodal. Therefore, chronic toxicity data for all species (Table S32) were used to calculate the ETVs consistent with Warne et al. (2018). The species sensitivity distribution and ecotoxicity threshold values for freshwater s-metolachlor data (Figure S30 and Table S33, respectively) were calculated using Burrlioz V2 (CSIRO 2016).


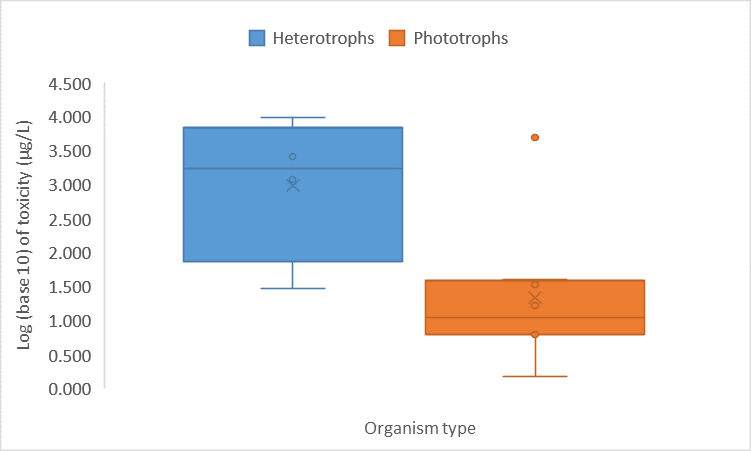


Figure S28. Box and whisker plots of S-metolachlor toxicity to freshwater phototrophic and heterotrophic organisms.


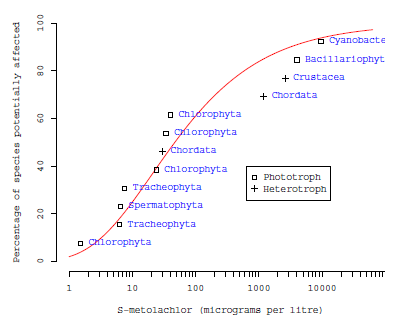


Figure S29. Species sensitivity distribution, generated by Burrlioz 2.0, for S-metolachlor to freshwater phototrophic and heterotrophic organisms to assist in assessing modality of the data.

Table S32. The ecotoxicity data used to calculate the ecotoxicity threshold values for S-metolachlor to freshwater species.

| **Species** | **Toxicity (µg/L)** |
| --- | --- |
| *Anabaena flos-aquae* | 9,600 |
| *Chlorella vulgaris* | 40 |
| *Navicula pelliculosa* | 4,000 |
| *Pimephales promelas* | 30 |
| *Raphidocelis subcapitata* | 1.5 |
| *Lemna gibba* | 7.6 |


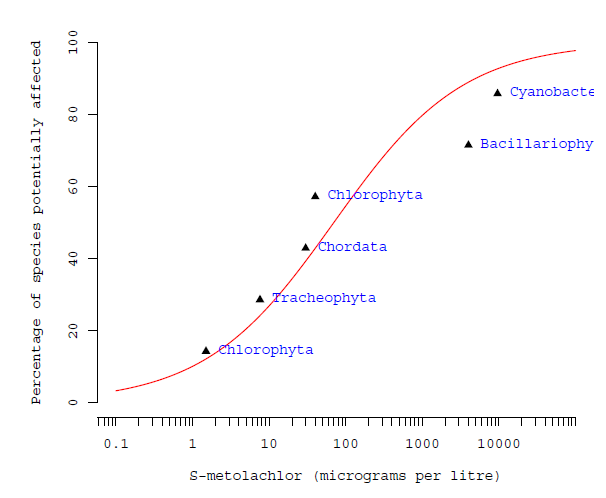


Figure S30. The species sensitivity distribution for chronic ecotoxicity data for S-metolachlor to all chronic freshwater species.

Table S33. The ecotoxicity threshold values derived for S-metolachlor to freshwater organisms.

| **Level of protection**  **(% species protected)** | **Concentration**  **(µg/L)** | **Concentration (µmol/L)** |
| --- | --- | --- |
| 99 | 0.0093 | 0.0000328 |
| 95 | 0.23 | 0.000810 |
| 90 | 0.98 | 0.00345 |
| 80 | 4.7 | 0.0166 |

### Tebuconazole in freshwater

Tebuconazole is a fungicide that affects fungi by preventing the formation of spores, preventing fungi from reproducing. It would therefore be expected that tebuconazole would be more toxic to fungi than other organisms. However, there were no toxicity data for fungi collated by the Swiss when deriving their water quality guideline (Oekotoxzentrum 2020) and therefore a comparison was not possible. There were data for phototrophs and heterotrophs so the sensitivity of these two groups were compared using the weight of evidence approach recommended by Warne et al. (2018). The coefficient of bimodality test yielded a value of 0.33 indicating the distribution of sensitivities is unimodal (as the minimum value to indicate bimodality is 0.55). The phototroph and heterotroph data were also compared using a box and whiskers plot (Figure S31) and a species sensitivity distribution (Figure S32). These indicated that there was an incomplete separation between the two groups. Given the distance of the bimodality coefficient value to the minimum cut-off value (i.e., 0.55) and the incomplete separation of the toxicity data for the phototrophs and heterotrophs it was decided that the distribution of all the available species was most likely unimodal. There were insufficient chronic toxicity data to derive ETVs, therefore, chronic and converted acute toxicity data for all species (Table S34) were used to calculate the ETVs consistent with Warne et al. (2018). The species sensitivity distribution and ETVs for freshwater tebuconazole data (Figure S32 and Table S35) were calculated using Burrlioz V2 (CSIRO 2016).


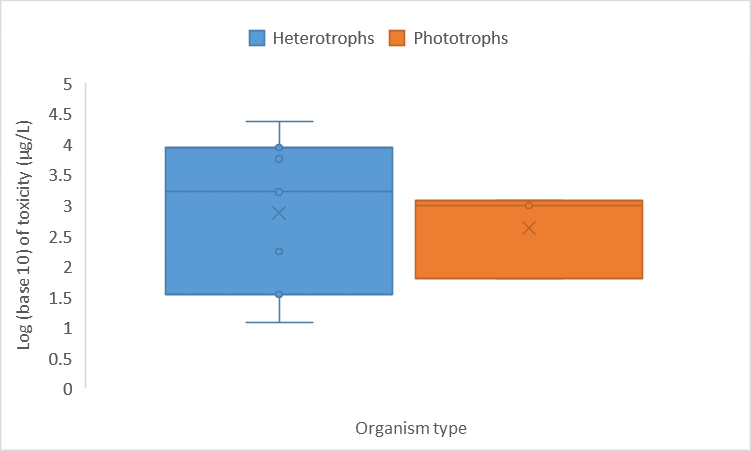


Figure S31. Box and whisker plot for tebuconazole freshwater toxicity to phototrophic and heterotrophic organisms.


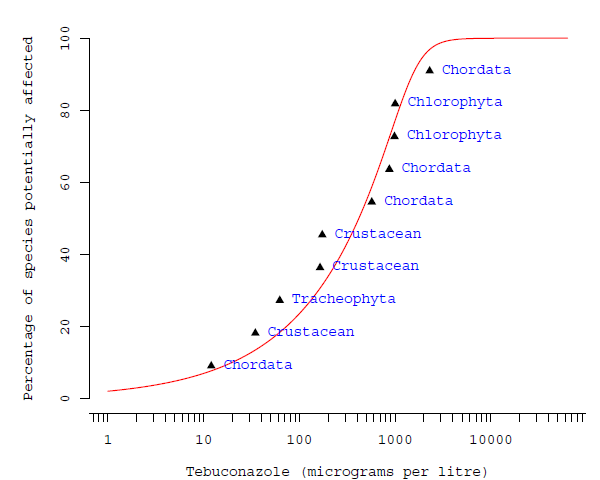


Figure S32. Species sensitivity distribution, generated by Burrlioz 2.0, for tebuconazole freshwater toxicity to phototrophic and heterotrophic organisms.

Table S34. The ecotoxicity data used to calculate the ecotoxicity threshold values for tebuconazole to freshwater species.

| **Species** | **Toxicity (µg/L)** |
| --- | --- |
| *Daphnia longispina* | 173.2 |
| *Desmodesmus subspicatus* | 1,000 |
| *Lemna gibba* | 62.3 |
| *Oncorhynchus mykiss* | 12 |
| *Pseudokirchneriella subcapitata* | 984 |
| *Danio rerio* | 2,294 |
| *Daphnia magna* | 34.6 |
| *Gammarus pulex (L.)* | 164 |
| *Lepomis macrochirus* | 570 |
| *Leuciscus idus* | 870 |

Table S35. The ecotoxicity threshold values derived for tebuconazole to freshwater organisms.

| **Level of protection**  **(% species protected)** | **Concentration**  **(µg/L)** | **Concentration**  **(µmol/L)** |
| --- | --- | --- |
| 99 | 0.25 | 0.0008 |
| 95 | 5.2 | 0.0169 |
| 90 | 20 | 0.0650 |
| 80 | 73 | 0.237 |

### Triadimenol in freshwater

The available chronic toxicity data for triadimenol from ECOTOX (US EPA, 2019b) and OPP (US EPA 2019c) are presented in Table S36. This permitted the derivation of a low reliability (Interim) DGV (Warne, 2001). Therefore, the toxicity value (which was a chronic value) of 0.145 mg/L (Daphnia magna) was divided by an assessment factor of 100 resulting in an ETV of 0.00145 mg/L or 1.45 µg/L. This equates to 0.0049 µmol/L. All DGVs derived by the assessment factor method are now classed as having an unknown reliability (Warne et al., 2018).

Table S36. Chronic toxicity data used to calculate the ecotoxicity threshold values for triadimenol to freshwater species.

| **Species** | **Taxa** | **Purity/Grade** | **Duration** | **Endpoint** | **Measure** | **Concentration (mg/L)** |
| --- | --- | --- | --- | --- | --- | --- |
| *Daphnia magna* | Crustacea | 92% | 21 d | Survival | NOEL | 0.145 |
| *Pseudokirchneriella subcapitata* | Green algae | 97.3% | 72 hr | N/A | NOEL | 0.85 |
| *Scenedesmus subspicatus* | Green algae | 94.9% | 96 hr | Abundance | NOEL | 0.32 |

### Trichlorfon in freshwater

Trichlorfon is an organophosphate insecticide that inhibits the enzyme acetylcholinesterase (AChE), so it is expected to be more toxic to arthropods (that includes insects and crustaceans) than non-arthropods. The sensitivity of arthropods and non-arthropods were compared following the weight of evidence approach recommended in Warne et al. (2018). The coefficient of bimodality test yielded a value of 0.35 indicating the distribution of sensitivities is unimodal (as the minimum value to indicate bimodality is 0.55). The arthropod and non-arthropod data were also compared using a box and whiskers plot (Figure S33), which indicated that the groups were not separated. Given the distance of the bimodality coefficient value to the minimum cut-off value (i.e., 0.55) and the incomplete separation of the toxicity data for the arthropod and non-arthropod data it was decided that the distribution was most likely unimodal. Therefore, toxicity data for all species (Table S37) were used to calculate the ecotoxicity thresholds consistent with Warne et al. (2018). The species sensitivity distribution and ecotoxicity threshold values for freshwater trichlorfon toxicity data (Figure S34 and Table S38, respectively) were calculated using Burrlioz V2 (CSIRO 2016).


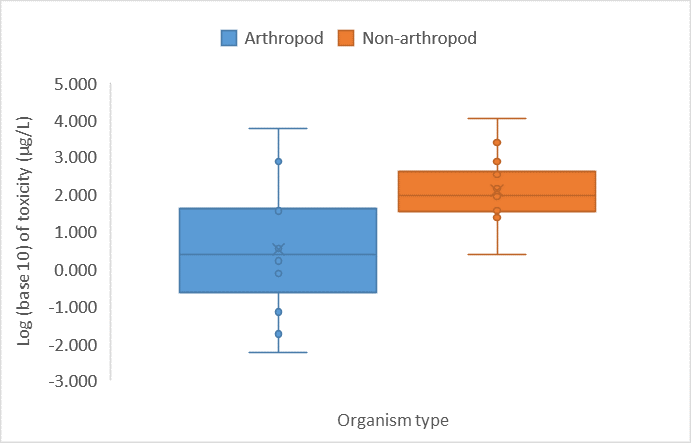


Figure S33. Box and whisker plot for trichlorfon freshwater toxicity to arthropod and non-arthropod organisms.

Table S37. The ecotoxicity data used to calculate the ecotoxicity threshold values for trichlorfon to freshwater species.

| **Species** | **Toxicity (µg/L)** |
| --- | --- |
| *Acroneuria pacifica* | 1.65 |
| *Claassenia sabulosa* | 2.20 |
| *Cyprinus carpio* | 6,026 |
| *Daphnia magna* | 0.006 |
| *Daphnia pulex* | 0.018 |
| *Gammarus lacustris* | 4.00 |
| *Gammarus pseudolimnaeus* | 51.9 |
| *Ictalurus melas* | 51.5 |
| *Ictalurus punctatus* | 88.0 |
| *Isogenus sp.* | 2.40 |
| *Lemna gibba* | 11,300 |
| *Lepomis macrochirus* | 95.9 |
| *Micropterus salmoides* | 345 |
| *Morone saxatilis* | 200 |
| *Oncorhynchus clarkii* | 37.5 |
| *Oncorhynchus mykiss* | 147 |
| *Penaeus duorarum* | 36.0 |
| *Pimephales promelas* | 790 |
| *Procambarus sp.* | 780 |
| *Pseudokirchneriella subcapitata* | 2,450 |
| *Pteronarcella badia* | 0.764 |
| *Pteronarcys californica* | 3.50 |
| *Salmo salar* | 30.0 |
| *Salvelinus fontinalis* | 24.0 |
| *Salvelinus namaycush* | 55.0 |
| *Simocephalus serrulatus* | 0.070 |
| *Skwala sp.* | 2.40 |

*
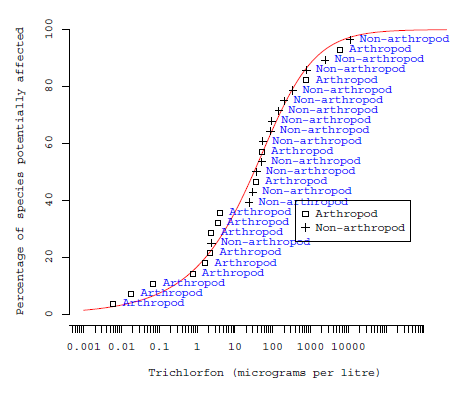
*

Figure S34. The species sensitivity distribution for chronic ecotoxicity data for trichlorfon to all available freshwater species.

Table S38. The ecotoxicity threshold values derived for trichlorfon to freshwater organisms.

| **Level of protection**  **(% species protected)** | **Concentration**  **(µg/L)** | **Concentration (µmol/L)** |
| --- | --- | --- |
| 99 | 0.00039 | 0.000002 |
| 95 | 0.034 | 0.000132 |
| 90 | 0.23 | 0.000893 |
| 80 | 1.7 | 0.00660 |

### Trifloxysulfuron sodium in freshwater

Trifloxysulfuron sodium is a sulfonylurea herbicide that inhibits the enzyme acetolactate synthase that synthesises amino acids, so it is expected to be more toxic to phototrophs than heterotrophs. The sensitivity of phototrophs and heterotrophs was compared following the weight of evidence approach recommended in Warne et al. (2018). The coefficient of bimodality test yielded a value of 0.16 indicating the distribution of sensitivities was unimodal (as the minimum value to indicate bimodality is 0.55). The phototrophs and heterotroph data were also compared using a box and whiskers plot (Figure S35), which indicated that the groups were not separated. Given the distance of the bimodality coefficient value to the minimum cut-off value (i.e., 0.55) and the incomplete separation of the toxicity data for the phototrophs and heterotroph data it was decided that the distribution was most likely unimodal. Therefore, toxicity data for all species (Table S39) were used to calculate the ecotoxicity thresholds consistent with Warne et al. (2018). The species sensitivity distribution and ETVs for freshwater trifloxysulfuron sodium data (Figure S36 and Table S40, respectively) were calculated using Burrlioz V2 (CSIRO 2016).


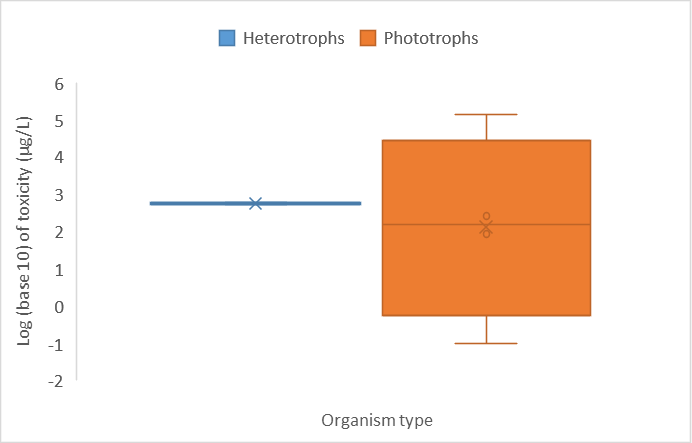


Figure S35. Box and whisker plot for trifloxysulfuron sodium freshwater toxicity to phototrophs and heterotrophs organisms.

Table S39. The ecotoxicity data used to calculate the ecotoxicity threshold values for trifloxysulfuron sodium to freshwater species.

| **Species** | **Toxicity (µg/L)** |
| --- | --- |
| *Daphnia magna* | 549 |
| *Oncorhynchus mykiss* | 604 |
| *Anabaena flos-aquae* | 266 |
| *Lemna gibba* | 0.102 |
| *Navicula pelliculosa* | 139,000 |
| *Pseudokirchneriella subcapitata* | 87.7 |


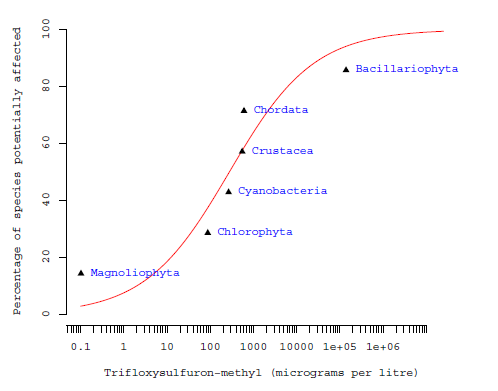


Figure S36. The species sensitivity distribution for chronic ecotoxicity data for trifloxysulfuron sodium to all available freshwater species.

Table S40. The ecotoxicity threshold values derived for trifloxysulfuron sodium to freshwater organisms.

| **Level of protection**  **(% species protected)** | **Concentration**  **(µg/L)** | **Concentration (µmol/L)** |
| --- | --- | --- |
| 99 | 0.0092 | 0.0000200 |
| 95 | 0.37 | 0.000806 |
| 90 | 2 | 0.00435 |
| 80 | 12 | 0.0261 |

### Trifluralin in freshwater

Trifluralin is a dinitroaniline herbicide that inhibits cell division and root and shoot growth. It is expected to be more toxic to phototrophs than heterotrophs. The sensitivity of phototrophs and heterotrophs was compared following the weight of evidence approach recommended in Warne et al. (2018). The coefficient of bimodality test yielded a value of 0.38 indicating the distribution of sensitivities is unimodal (as the minimum value to indicate bimodality is 0.55). The phototrophs and heterotroph data were also compared using a box and whiskers plot (Figure S37), which indicated that the groups were not separated. Given the distance of the bimodality coefficient value to the minimum cut-off value (i.e., 0.55) and the extensive overlap of the toxicity data for the phototrophs and heterotroph data it was decided that the distribution was most likely unimodal. Therefore, chronic NOEC/EC10 and chronic LOEC/EC50 toxicity data for all species (Table S41) were used to calculate the ecotoxicity thresholds consistent with Warne et al. (2018). The species sensitivity distribution and ecotoxicity threshold values for freshwater trifluralin data (Figure S38 and Table S42, respectively) were calculated using Burrlioz V2 (CSIRO 2016).

Figure S37. Box and whisker plots for trifluralin freshwater toxicity to phototrophs and heterotrophs organisms.

Table S41. The ecotoxicity data used to calculate the ecotoxicity threshold values for trifluralin to freshwater species.

| **Species** | **Toxicity (µg/L)** |
| --- | --- |
| *Anabaena flos-aquae* | 273 |
| *Chironomus riparius* | 2,150 |
| *Daphnia magna* | 11.0 |
| *Hyalella azteca* | 6,260 |
| *Oncorhynchus mykiss* | 2.18 |
| *Pimephales promelas* | 0.75 |
| *Lemna gibba* | 9.9 |
| *Navicula pelliculosa* | 7.5 |


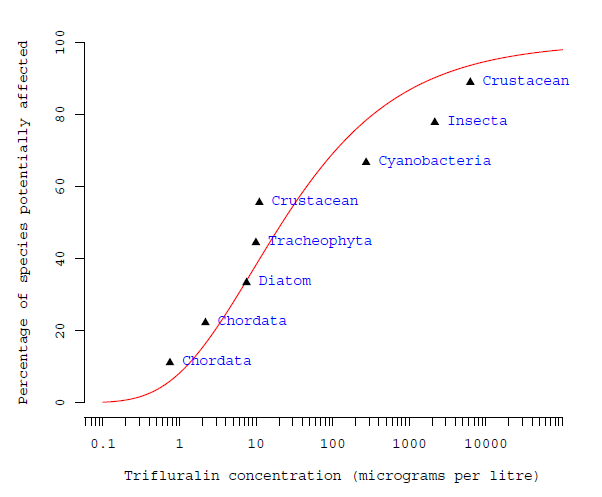


Figure S38. The species sensitivity distribution for chronic NOEC/EC10 and chronic LOEC/EC50 ecotoxicity data for trifluralin to freshwater species.

Table S42. The ecotoxicity threshold values derived for trifluralin to freshwater organisms.

| **Level of protection**  **(% species protected)** | **Concentration**  **(µg/L)** | **Concentration (µmol/L)** |
| --- | --- | --- |
| 99 | 0.23 | 0.000686 |
| 95 | 0.64 | 0.00191 |
| 90 | 1.2 | 0.00358 |
| 80 | 2.9 | 0.00865 |

### Trinexapac-ethyl in freshwater

Trinexapac-ethyl is a herbicide (a growth regulator) that inhibits the synthesis of gibberellic acid (GA1) in plants which in turn stops cell elongation in plants during the vegetative stage. As such it is expected that trinexapac-ethyl would be more toxic to phototrophs than to heterotrophs. The sensitivity of phototrophs and heterotrophs was compared following the weight of evidence approach recommended in Warne et al. (2018). The coefficient of bimodality test yielded a value of 0.51 indicating the distribution of sensitivities is unimodal (as the minimum value to indicate bimodality is 0.55). The phototrophs and heterotroph data were also compared using a box and whiskers plot (Figure S39), which indicated that the groups were not separated. Given the bimodality coefficient and the extensive overlap of the toxicity data for the phototrophs and heterotroph data it was decided that the distribution was most likely unimodal. Therefore, chronic NOEC/EC10 toxicity data for all species (Table S43) were used to calculate the ecotoxicity thresholds consistent with Warne et al. (2018). The species sensitivity distribution and ecotoxicity threshold values for freshwater trifluralin data (Figure S40 and Table S44, respectively) were calculated using Burrlioz V2 (CSIRO 2016).


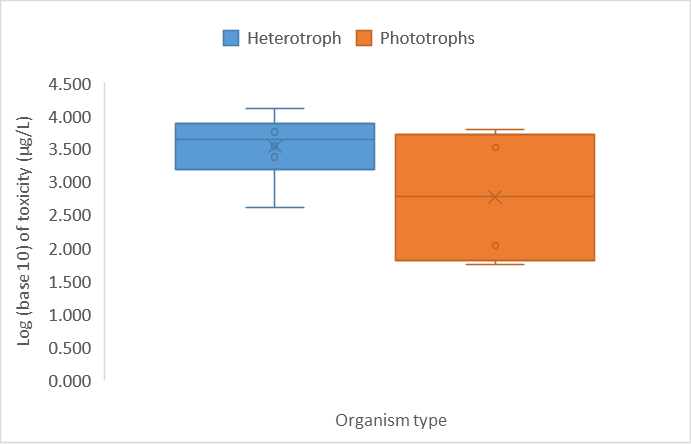


Figure S39. Box and whisker plot for trinexapac-ethyl freshwater toxicity to phototrophs and heterotrophs organisms.

Table S43. The ecotoxicity data used to calculate the ecotoxicity threshold values for trinexapac-ethyl to freshwater species.

| **Species** | **Toxicity (µg/L)** |
| --- | --- |
| *Anabaena flos-aquae* | 110 |
| *Daphnia magna* | 2,400 |
| *Navicula pelliculosa* | 6,200 |
| *Pimephales promelas* | 410 |
| *Pseudokirchneriella subcapitata* | 3,305 |


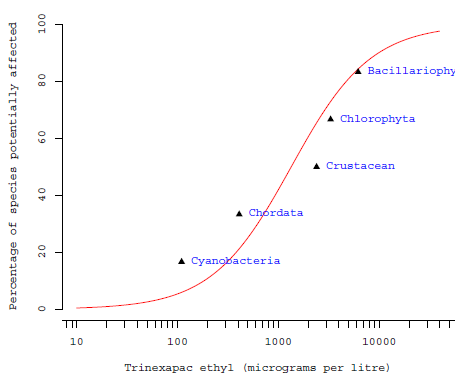


Figure S40. The species sensitivity distribution for chronic NOEC/EC10 ecotoxicity data for trinexapac-ethyl to freshwater species.

Table S44. The ecotoxicity threshold values derived for trinexapac-ethyl to freshwater organisms.

| **Level of protection**  **(% species protected)** | **Concentration**  **(µg/L)** | **Concentration (µmol/L)** |
| --- | --- | --- |
| 99 | 21 | 0.0832 |
| 95 | 93 | 0.369 |
| 90 | 183 | 0.725 |
| 80 | 383 | 1.518 |

Figure S41: Plots of ranked measure of effect for maximum vs minimum application rate, maximum vs average application rate and average vs minimum application rate.

Figure S42: Linear relationship between OPERA modelled log K_OC_ values (US EPA 2019a) and experimental log K_OC_ values from the Pesticide Property Database (University of Hertfordshire 2013).


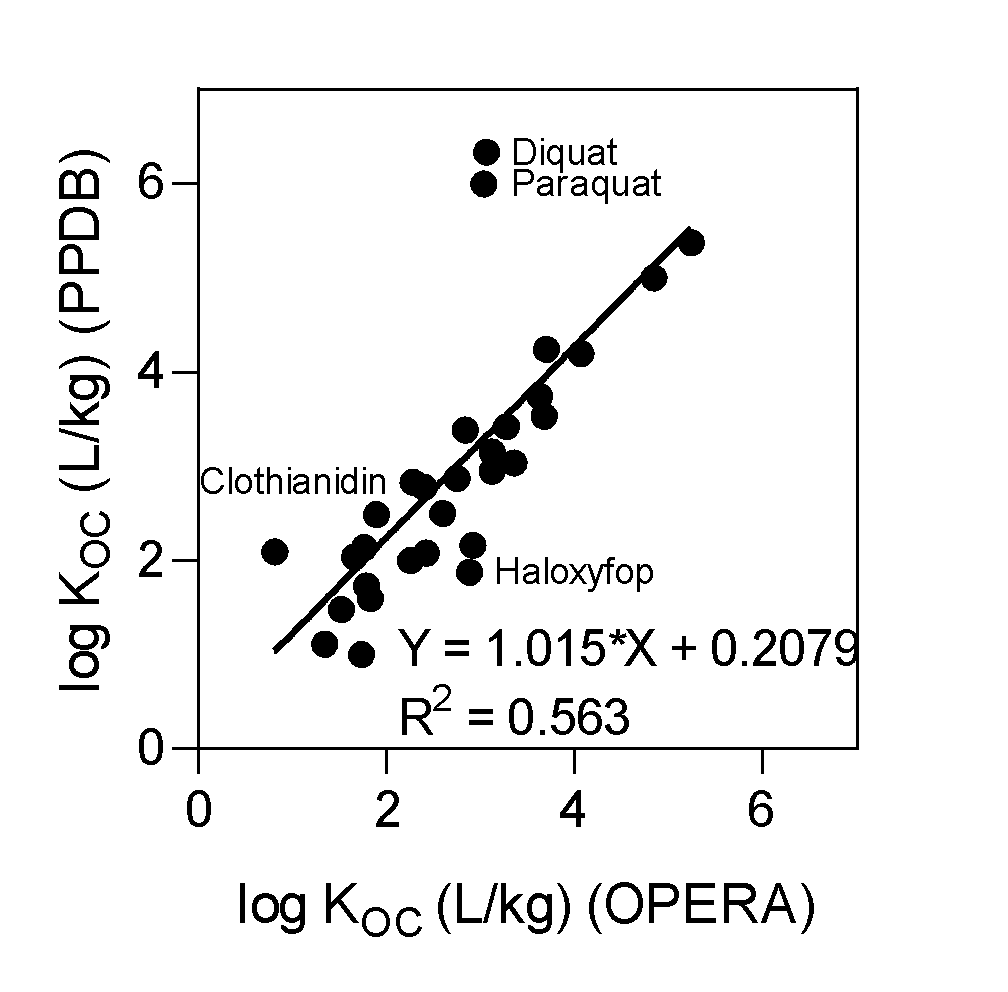


Figure S43: Plot of the measure of effect and the measure of mobility and persistence for all the herbicide active ingredients registered for application to sugar cane. The closed symbols were derived using PC95 and the open symbols were derived using PC99. Active ingredients with DGVs or ETVs with moderate, high or very high reliability are indicated in italics, while active ingredients with DGVs or ETVs with unknown, very low or low reliability are indicated in normal font.


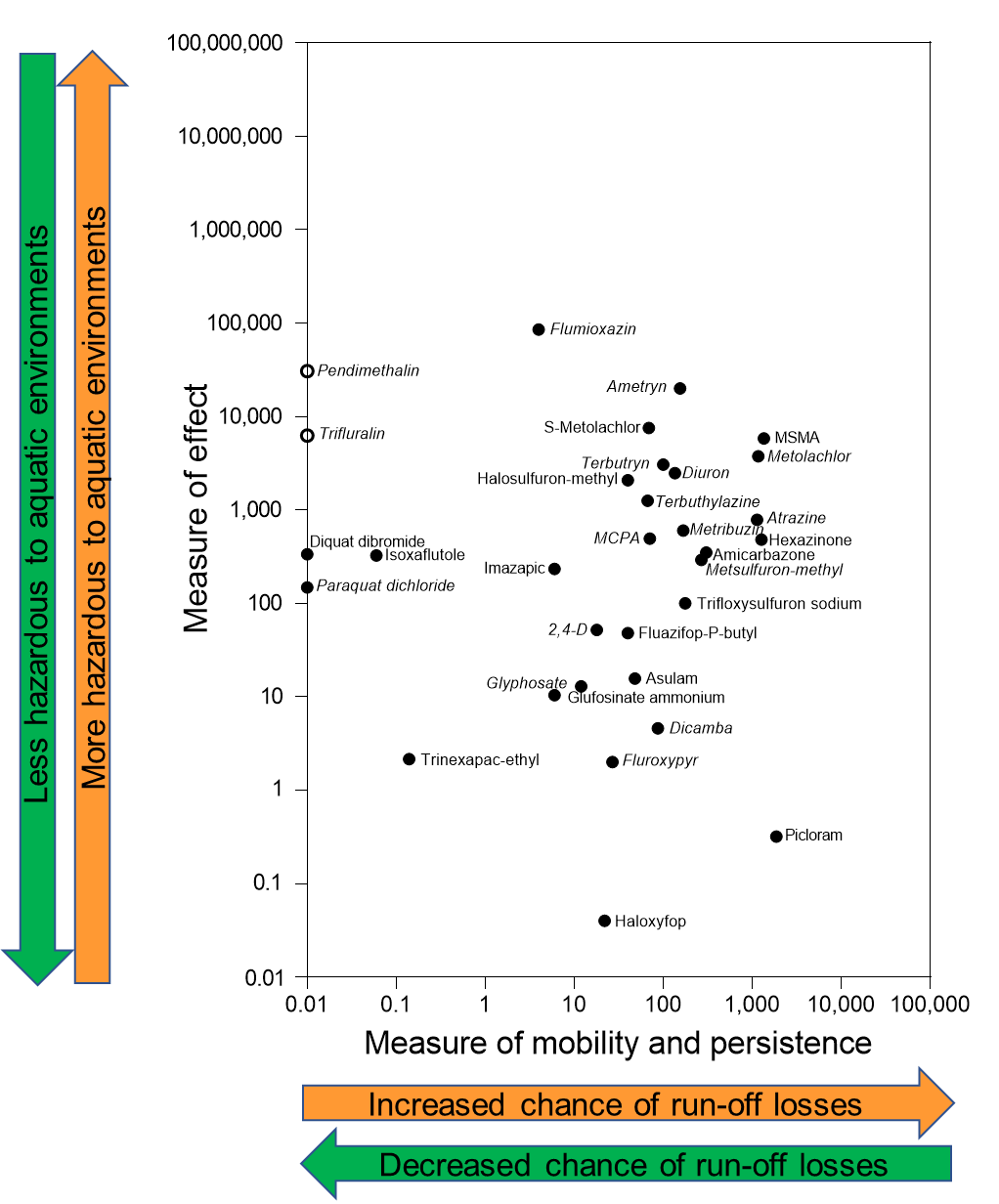


Figure S44: Plot of the measure of effect and the measure of mobility and persistence for all the insecticide active ingredients registered for application to sugar cane. The closed symbols were derived using PC95 and the open symbols were derived using PC99. Active ingredients with DGVs or ETVs with moderate, high or very high reliability are indicated in italics, while active ingredients with DGVs or ETVs with unknown, very low or low reliability are indicated in normal font. NB: both slow release (SR) and liquid (L) imidacloprid were included.


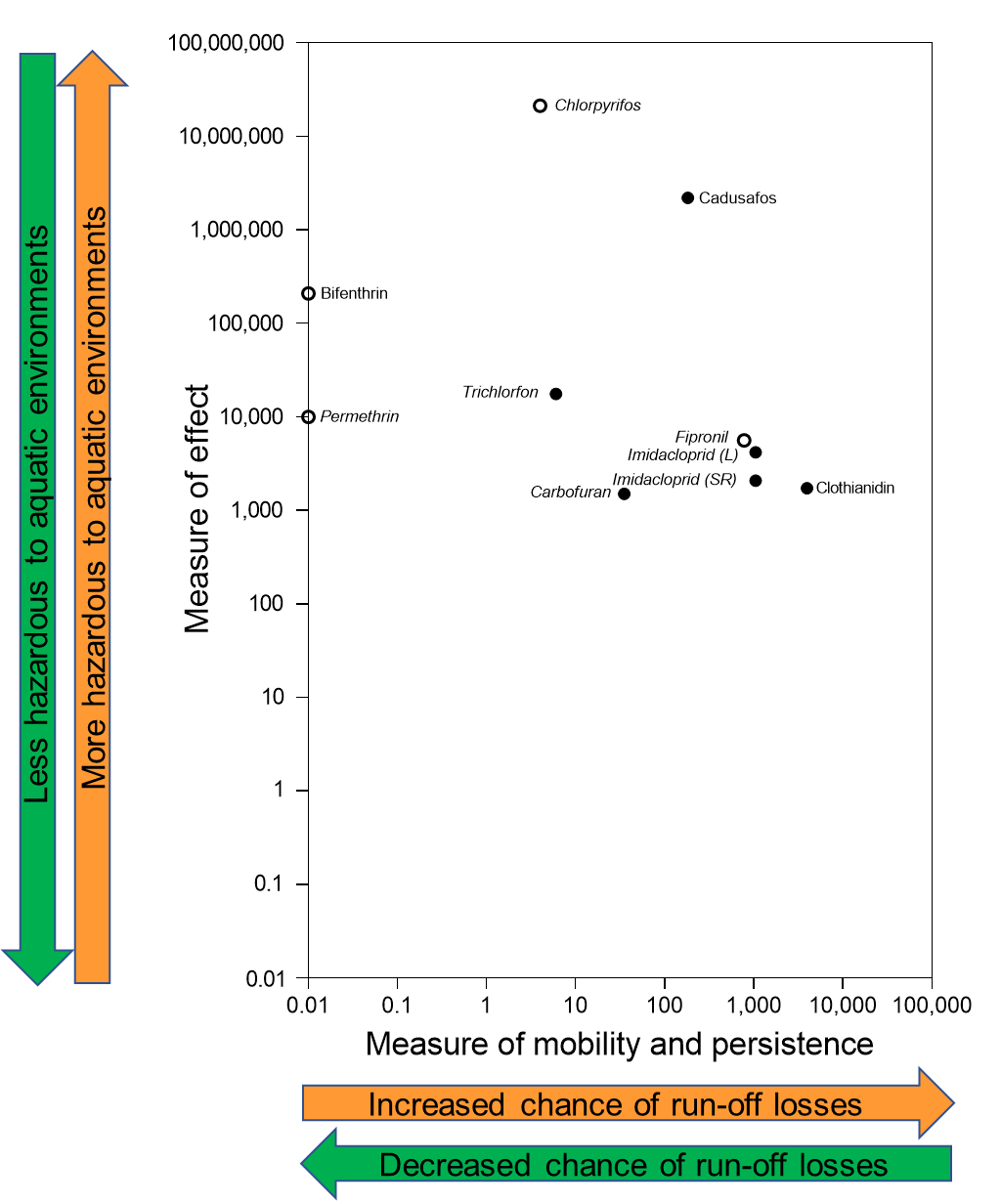


Figure S45: Plot of the measure of effect and the measure of mobility and persistence for all the fungicide active ingredients registered for application to sugar cane. Active ingredients with DGVs or ETVs with moderate, high or very high reliability are indicated in italics, while active ingredients with DGVs or ETVs with unknown, very low or low reliability are indicated in normal font.


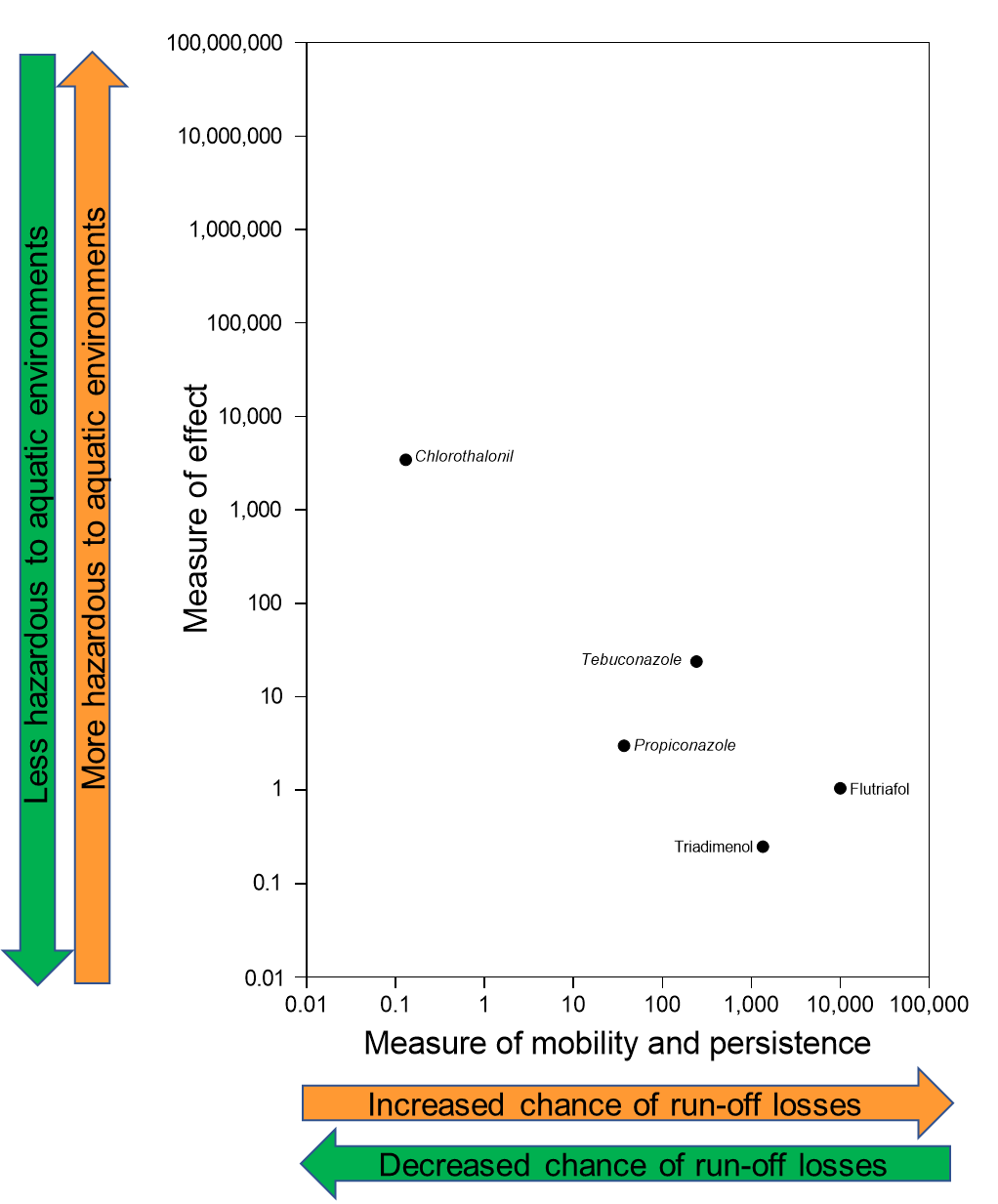


**References**

ANZECC and ARMCANZ (2000) National Water Quality Management Strategy, Paper No. 4, Australian and New Zealand Guidelines for Fresh and Marine Water Quality, Volume 1, The Guidelines (Chapters 1–7). , Australian and New Zealand Environment and Conservation Council and Agriculture and Resource Management Council of Australia and New Zealand, Canberra, Australia.

APVMA (2019) Public Chemical Registration Information System Search (PubCRIS). Available from: <https://portal.apvma.gov.au/pubcris>

CSIRO (2016) Burrlioz - statistical software package to generate trigger values for local conditions within Australia. CSIRO. Available from: <https://research.csiro.au/software/burrlioz/>

King OC, Smith RA, Mann RM, Warne MSJ (2017a) Proposed aquatic ecosystem protection guideline values for pesticides commonly used in the Great Barrier Reef catchment area: Part 1–2, 4-D, Ametryn, Diuron, Glyphosate, Hexazinone, Imazapic, Imidacloprid, Isoxaflutole, Metolachlor, Metribuzin, Metsulfuron-methyl, Simazine and Tebuthiuron, Department of Science, Information Technology and Innovation. Brisbane, Queensland, Australia

King OC, Smith RA, Warne MSJ, Frangos JS, Mann RM (2017b) Proposed aquatic ecosystem protection guideline values for pesticides commonly used in the Great Barrier Reef catchment area: Part 2 - Bromacil, Chlorothalonil, Fipronil, Fluometuron, Fluroxypyr, Haloxyfop, MCPA, Pendimethalin, Prometryn, Propazine, Propiconazole, Terbutryn, Triclopyr and Terbuthylazine, Department of Science, Information Technology and Innovation. Brisbane, Queensland, Australia.

National Center for Biotechnology Information (2019) PubChem Database. Available from: <https://pubchem.ncbi.nlm.nih.gov/>

Oekotoxzentrum (2020) Proposals for Acute and Chronic Quality Standards. Available from: <https://www.ecotoxcentre.ch/expert-service/quality-standards/proposals-for-acute-and-chronic-quality-standards/>

University of Hertfordshire (2013) The Pesticide Properties Data Base (PPDB). Developed by the Agriculture & Environment Research Unit (AERU), University of Hertfordshire, 2006–2013. Available from: <http://sitem.herts.ac.uk/aeru/ppdb/en/Reports/27.htm>.

US EPA (2012) Estimation Programs Interface Suite for Windows, v4.1, United States Environmental Protection Agency, Washington, DC, USA

US EPA (2019a) CompTox Chemistry Dashboard. Available from: <https://comptox.epa.gov/dashboard>

US EPA (2019b) ECOTOX Knowledgebase. Available from: <https://cfpub.epa.gov/ecotox/>

US EPA (2019c) OPP Pesticide Ecotoxicity Database. Available from: <https://ecotox.ipmcenters.org/index.cfm?menuid=7>

Walters J (1999) Environmental fate of 2,4-dichlorophenoxyacetic acid, Department of Pesticide Regulations, Environmental Monitoring and Pest Management, Sacramento, California, CA 95814-3510. Available from: <http://www.cdpr.ca.gov/docs/emon/pubs/fatememo/24-d.pdf>

Warne MSJ (2001) Derivation of the Australian and New Zealand water quality guidelines for toxicants. Australas J Ecotoxicol 7:123-136.

Warne MSJ, Batley GE, van Dam RA, Chapman JC, Fox DR, Hickey CW, Stauber JL (2018) Revised Method for Deriving Australian and New Zealand Water Quality Guideline Values for Toxicants – update of 2015 version. Prepared for the revision of the Australian and New Zealand Guidelines for Fresh and Marine Water Quality, Australian and New Zealand Governments and Australian state and territory governments, Canberra, Australia

Excel spreadsheet version of the Pesticide Decision Support Tool

Warne et al. ESM ESPR May 2023
